# Climate induced human demographic and cultural change in northern Europe during the mid-Holocene

**DOI:** 10.1038/s41598-017-14353-5

**Published:** 2017-11-10

**Authors:** L. Warden, M. Moros, T. Neumann, S. Shennan, A. Timpson, K. Manning, M. Sollai, L. Wacker, K. Perner, K. Häusler, T. Leipe, L. Zillén, A. Kotilainen, E. Jansen, R. R. Schneider, R. Oeberst, H. Arz, J. S. Sinninghe Damsté

**Affiliations:** 1 NIOZ Royal Netherlands Institute for Sea Research, Department of Marine Microbiology and Biogeochemistry, and Utrecht University, 1790 AB Den Burg, Texel, The Netherlands; 2 Leibniz Institute for Baltic Sea Research, Departments of Marine Geology and Physical Oceanography, Warnemünde, Germany; 3 Institute of Archaeology, University College London, 31-34 Gordon Square, London, WC1H 0PY UK; 4 Department of Genetics, Evolution and Environment, University College London, Gower Street, London, WC1E 6BT UK; 5 Department of Geography, King’s College London, London, WC2R 2LS UK; 6 Laboratory of Ion Beam Physics, ETH Zürich, Otto-Stern-Weg 5, 8093 Zürich, Switzerland; 7 Geological Survey of Sweden, Department of Marine Geology, Box 670, 751 28 Uppsala, Sweden; 8 Geological Survey of Finland, P.O. Box 96, 02151 Helsinki, Finland; 9 Bjerknes Centre for Climate Research/Department of Earth Science, University of Bergen Allégaten 70, 5007 Bergen, Norway; 10 Insitute of Geosciences, Kiel University, Ludewig-Meyn Strasse 10, 24118 Kiel, Germany; 11 Institute of Baltic Sea Fisheries, Thünen Institute, Alter Hafen Süd 2, 18069 Rostock, Germany; 12 Utrecht University, Faculty of Geosciences, P.O. Box 80.021, 3508 TA Utrecht, The Netherlands

## Abstract

The transition from hunter-gatherer-fisher groups to agrarian societies is arguably the most significant change in human prehistory. In the European plain there is evidence for fully developed agrarian societies by 7,500 cal. yr BP, yet a well-established agrarian society does not appear in the north until 6,000 cal. yr BP for unknown reasons. Here we show a sudden increase in summer temperature at 6,000 cal. yr BP in northern Europe using a well-dated, high resolution record of sea surface temperature (SST) from the Baltic Sea. This temperature rise resulted in hypoxic conditions across the entire Baltic sea as revealed by multiple sedimentary records and supported by marine ecosystem modeling. Comparison with summed probability distributions of radiocarbon dates from archaeological sites indicate that this temperature rise coincided with both the introduction of farming, and a dramatic population increase. The evidence supports the hypothesis that the boundary of farming rapidly extended north at 6,000 cal. yr BP because terrestrial conditions in a previously marginal region improved.

## Introduction

The transition from foraging to farming provided the basis for a massive increase in food production and human population density, which in turn drove the accumulation of technology and specialization, ultimately enabling the establishment of modern day societies^1^. The reasons and mechanisms for this Neolithic Revolution occurring at different times in different places continues to be debated. In Northern Europe cereal agriculture and animal husbandry did not begin until ca. 6,000 cal. yr BP, i.e. 1,500 years later than in neighboring populations to the south^2–6^. It has been proposed that foraging societies were successful in relying largely on the marine resources from the Baltic Sea and had no incentive to adopt an agrarian lifestyle^5^, which required more intensive labor with no immediate increase in living standards^7^. This leaves the question of why the transformation did eventually occur at 6,000 cal. yr BP. It has been suggested that climate change may have been responsible for the late onset of farming in the northern European regions^2^; however, due to the lack of high resolution climate reconstructions the effects of environmental change on human societies in these regions have been unclear. In order to examine this at a fine temporal scale, we employed an approach combining sediment proxy studies with 3D ecosystem modeling to reconstruct climate conditions in northern Europe and environmental conditions in the Baltic Sea and compare this with human population and subsistence changes from archeological sites from the region.

## Results and Discussion

**Figure 1:**
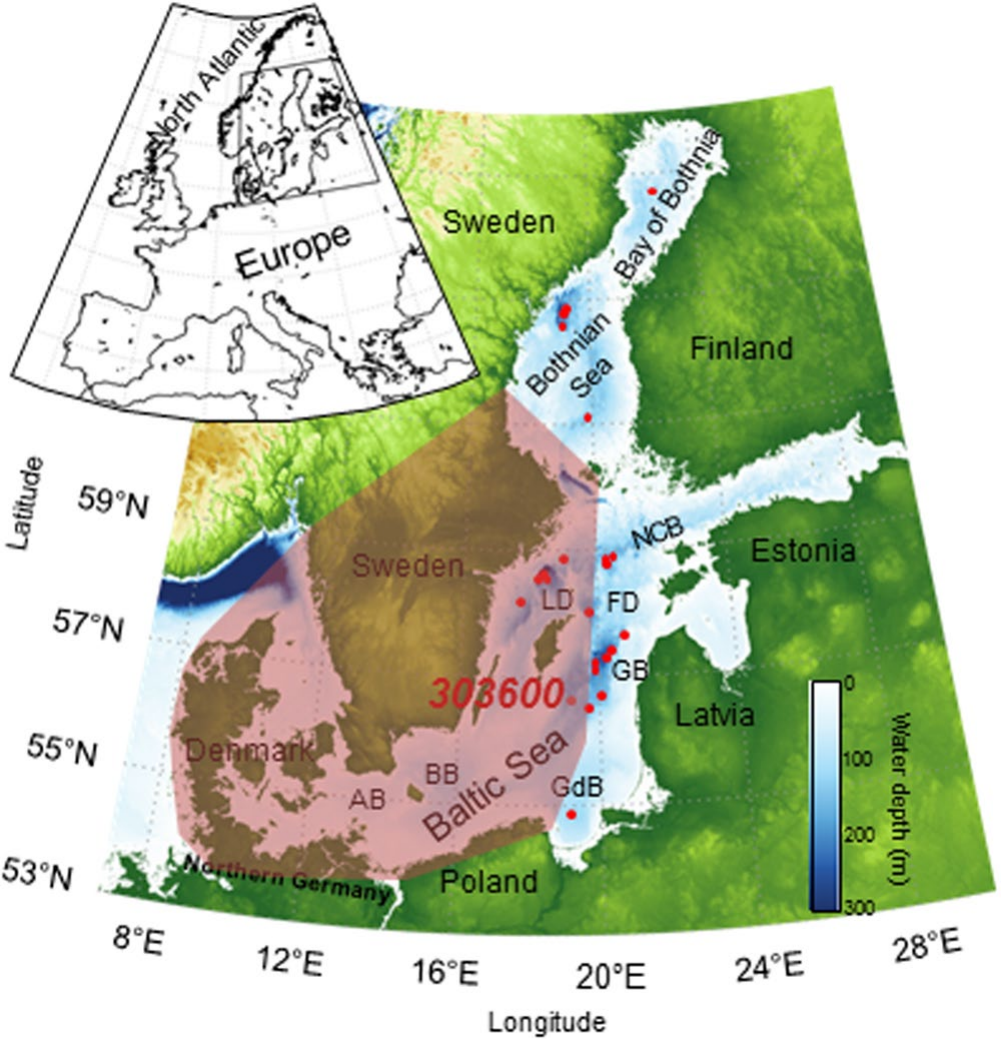
Sediment coring stations and archaeological sites in the Baltic Sea region. Location of the sediment study sites are indicated with red dots with the location of the key-site 303600 labeled. The inset shows the location of the Baltic Sea area in Europe. Radiocarbon dates for the population proxy were taken from archaeological sites within the brown polygon. The Baltic Sea´s modern bathymetry is shown along with the Baltic Sea basins: AB – Arkona Basin, BB – Bornholm Basin, GdB – Gdansk Basin, GB – Gotland Basin, FD – Fårö Deep, LD – Landsort Deep, NCB – Northern Central Basin. The maps were created using the software package GrADS 2.1.1.b0 (http://cola.gmu.edu/grads/), using published bathymetry data^42,43^.

**Figure 2:**
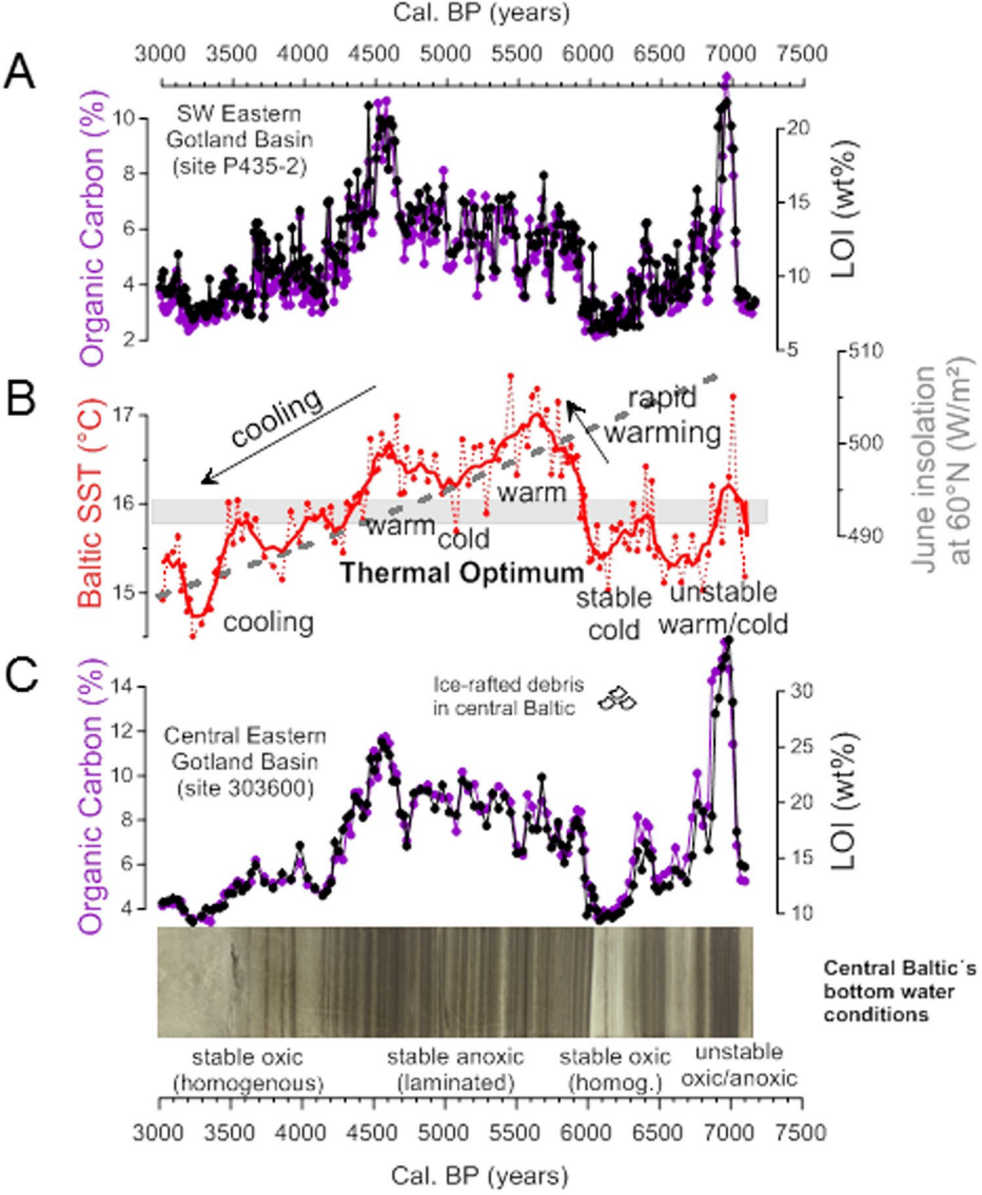
Regional climatic and environmental changes in the Baltic Sea from 7,100 to 3,000 cal. yr BP. (**A** and **C)** Loss on ignition (LOI, black) and organic carbon content (purple) data from two key-sites P435-2 and 303600 vs. age. Sediments with high organic carbon content are dark and laminated, corresponding to periods of bottom water anoxia. Comparison of the two records shows that they are highly correlated, allowing the age-depth model for P435-2 to be adapted to provide chronology for 303600. (**B**) Summer SST record for Gotland Basin site 303600 based on TEX_86_ palaeothermometry. The solid red line plots the 5-point rolling mean. June insolation at 60°N (in W m^−2^) is plotted as a grey dashed line. Horizontal grey bar marks the 16 °C threshold temperature for the occurrence of cyanobacterial blooms. Note, anoxic conditions of bottom waters (i.e. laminated sediments and elevated LOI values) occurred when temperatures were above this threshold temperature. The rapid warming with the sudden appearance/spread of anoxic conditions occurred at 6,000 cal. yr BP and is followed by a regional Thermal Optimum. The general temperature decline/cooling follows the summer insolation decrease finally leading to stable oxic bottom water conditions. A strong cold phase between 6,300 and 6,000 cal. yr BP is also evident from an increase in coarse mineral fraction (ice rafted debris) in central Baltic Sea sediments.

We examined key-sedimentary records from the central Baltic Sea (Fig. 1) from 7,100-3,000 cal. yr BP (Table 1). Complementary information for the entire Baltic was obtained from a number of other sediment cores (Figs 1, S1 and S2; Table 1). Our detailed age-depth model (Fig. S3) was developed based on radiocarbon dating of benthic foraminifera (Table S1) that are present in sediments from the deepest part of the Gotland Basin when inflowing saline waters from the North Sea reach the basin. This age-depth model was projected onto shallow water sites using LOI and/or organic carbon downcore profiles (see methods). The deep sites are, however, influenced by lateral transport of fine-grained material and, therefore, not suitable for sediment proxy reconstruction of past changes in *in-situ* surface water conditions. Therefore, a core from site 303600 (175 m water depth), located outside the inflowing water pathway on the western side of the eastern Gotland Basin (Fig. S1), was selected for determining the palaeotemperature record. This core was analyzed in high resolution using the TEX_86_-paleothermometer, a proxy for reconstructing past sea surface temperature (SST) based on the distribution of Thaumarchaeotal membrane lipids^8^. We applied a local calibration of TEX_86_ to reconstruct summer SST in the Baltic Sea^9^. Our record shows that summer SST varied between 14.5-17.5 °C during the study period (Fig. 2B). A prolonged unstable but generally colder phase is terminated at 6,000 cal. yr BP by a distinct and rapid warming phase (Fig. 2B). Two SST maxima at 5,600 and 4,500 cal. yr BP are evident during this regional Holocene Thermal Optimum lasting from ca. 5,900 to 4,400 cal. yr BP. Throughout the mid- to late-Holocene the general summer SST trend declines, following summer solar insolation (Fig. 2B).

**Table 1:** Details on the core sites studied located in almost all basins of the Baltic Sea. The location (see Figure S1 for a map), present-day and estimated paleo-water depths (6,500 cal. yr BP) are indicated, along with cruises and years of sampling of sites. Key-sites for chronology and proxy studies are marked in bold font. Loss on ignition (LOI) data are available from all sites. LOI data from cores marked with (X) are shown in Figs S2–S4.

Site	latitude	longitude	water depth (m)	paleo water depth (m)	Research vessel/cruise	Sampling Year	LOI data
*Gdansk Basin*							
P475-12-6	54°49.38 ′N	19°11.16′E	105	101	Poseidon 475	2014	X
*Gotland Basin*							
**303600**	56°55.01′N	19°19.99′E	170	190	Prof. Albrecht Penck	2009	X
**370540-6 (−7)**	57°17.01′N	20°07.25′E	243	264	Aranda	2009	X^a^/X
349110					Maria S. Merian	2008	X
MSM16/1-052					Maria S. Merian 16/1	2010	X
**P435-2-1**	57°06.33′N	19°50.77′E	218	232	Poseidon 435	2012	X
303620-3					Poseidon	2005	X
372740	57°23.10′N	20°15.50′E	232	255	Maria S. Merian 12/4	2009	X^a^
370530					Aranda	2009	X^a^
211640-7					Kotzov	1997	X
303630-3	57°00.37′N	19°50.08′E	185	199	Poseidon	2005	X
303640-6	56°43.66′N	19°57.49′E	153	165	Poseidon	2005	X
303650-5	56°30.05′N	19°40.46′E	141	155	Poseidon	2005	X
MSM16/1-057	57°38.88′N	20°38.75′E	139	165	Maria S. Merian 16/1	2010	X
*Farö Deep*							
F80	58°00.00′N	19°53.81′E	181	225	Aranda	2009	X^b^
*Northern Central Basin*							
243060	58°46.90′N	20°15.30′E	190	211	Poseidon 282	2002	X
243070	58°46.10′N	20°15.30′E	195	211	Poseidon 282	2002	X
349140	58°48.99′N	20°25.07′E	189	222	Maria S. Merian	2008	X
P435-17	58°42.75′N	20°14.68′E	210	243	Poseidon 435	2012	X
*Landsort Deep*							
349210	58°33.51′N	18°13.83′E	258	314	Maria S. Merian	2008	X
M86/1-33	58°21.90′N	17°50.04′E	101	144	Meteor M86/1	2011	X
M86/1-37	58°40.39′N	18°31.07′E	250	323	Meteor M86/1	2011	X
M86/1-38	58°35.84′N	18°28.03′E	179	236	Meteor M86/1	2011	X
M86/1-39	58°58.42′N	19°14.43′E	104	154	Meteor M86/1	2011	X
*Bothnian Sea*							
P435-10	62°52.16′N	19°02.55′E	214	365	Poseidon 435	2012	X^c^
P435-11	62°50.70′N	18°53.27′E	206	357	Poseidon 435	2012	X
P435-12	62°52.78′N	18°55.25′E	178	328	Poseidon 435	2012	X^c^
P435-13	62°35.18′N	18°58.13′E	215	365	Poseidon 435	2012	X
P435-14	61°03.98′N	19°43.13′E	141	208	Poseidon 435	2012	X^c^
*Bay of Bothnia*							
MSM16/1-93	64°42.00′N	22°03.72′E	130	211	Maria S. Merian 16/1	2006	X^c^

^a^LOI record previously published^36^; ^b^LOI record previously published^49^, ^c^LOI record previously published^50^.

The present-day Baltic Sea is persistently stratified with anoxic bottom waters in the deep basins and oxygen-depleted (hypoxic) waters in coastal areas (so-called dead-zones^10,11^). Summer SST is thought to have controlled hypoxia in the Baltic Sea over the last 1000 yrs through various feedback loops^9,12^. A comparison of SST, the total organic carbon (TOC) content and the degree of lamination of the sediments indicates that the critical 16 °C temperature threshold for the occurrence of severe hypoxia proposed for the last 1000 yrs^9^, also acted earlier in the Holocene (Fig. 2). The marked rapid warming phase at 6,000 cal. yr BP co-occurs with the onset of bottom water hypoxia (Fig. 2). This intensification of hypoxia at 6,000 cal. yr BP is evident in the sedimentary records from the entire Paleo-Baltic (Fig. S4). The stable hypoxic conditions of the Thermal Optimum phase, when summer SST is persistently >16 °C, are widespread in the entire Baltic Sea (Fig. S4) and terminated by a marked cooling after 4,500 cal. yr BP when summer SST dropped below 16 °C (Fig. 2).

**Figure 3:**
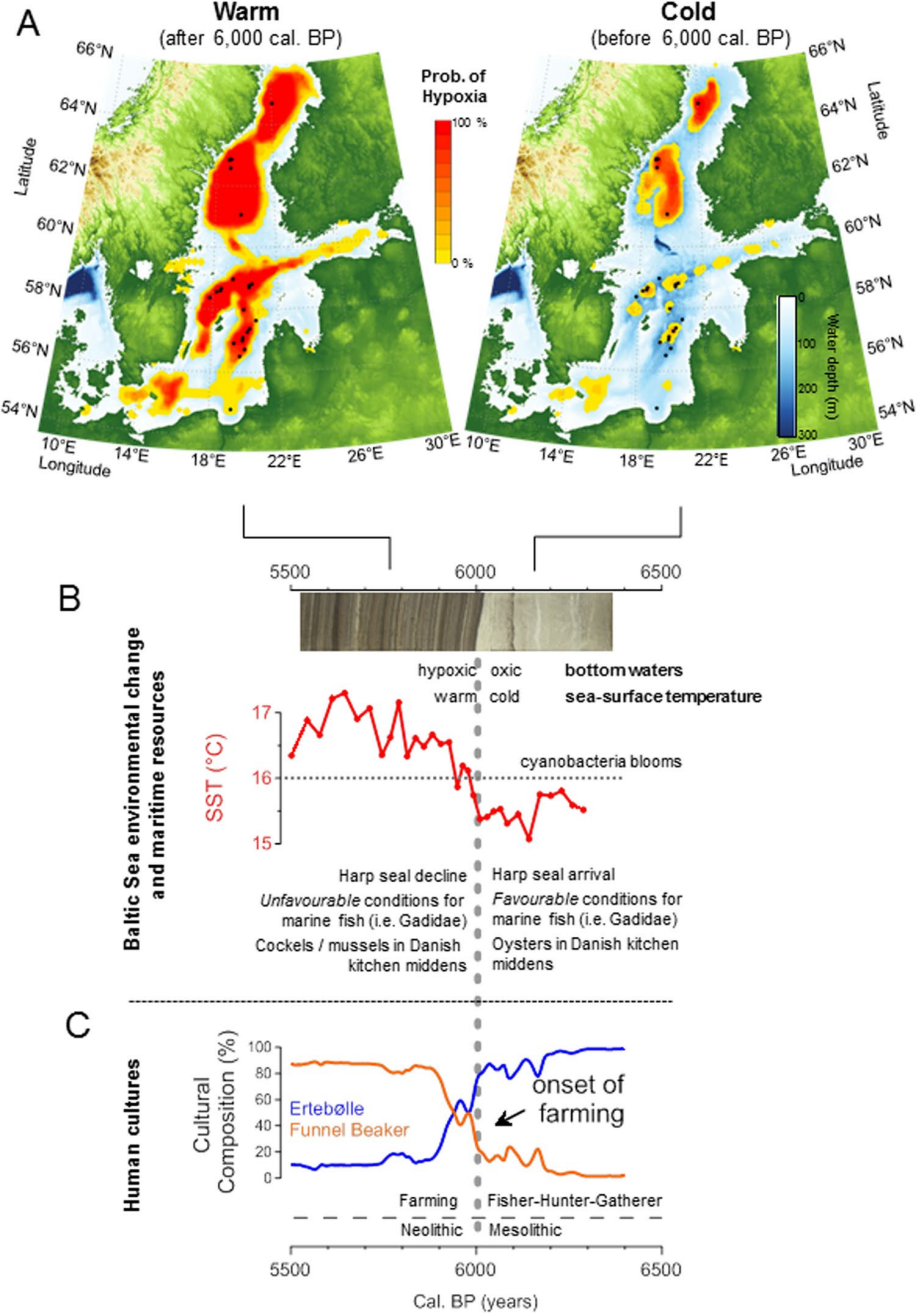
The 6,000 cal. yr BP event: Ecosystem modelling, palaeoclimate and archaeology. Increasing SST after 6,000 cal. yr BP likely improved conditions for farming in the region and thus prompted the migration of the Funnel Beaker culture north. (**A**) Using 3D Ecosystem modelling based on paleobathymetry, the extent of hypoxia in the Baltic Sea is shown for the cold phase (right panel) before 6,000 cal. yr BP and for the warm phase (left panel) after 6,000 cal. yr BP. Note the remarkable spreading of hypoxic areas to the whole basin after 6,000 cal. yr BP, suggesting i) the regional wide scale of the climatic changes and ii) a close relationship between SST rise and spread of anoxia (see SI), which may have had consequences for marine life, therefore affecting the hunter-gatherer-fisher society. (**B**) Regional environmental changes in the Baltic Region at this time. The sequence from non-laminated to laminated sediments along with the significant increase in organic carbon content reveals that the development of anoxic bottom water conditions which is related to the substantial increase in SST. (**C**) Population density based on SPD. Note the population growth rate increases at the boundary between the Meso- and Neolithic. The maps were created using the software package GrADS 2.1.1.b0 (http://cola.gmu.edu/grads/), using published bathymetry data^42,43^.

Our analysis using a coupled hydrodynamic-biogeochemical model applying a reconstructed paleo-bathymetry (Fig. S5) and forced by a delta change approach (Fig. S6) suggests that the SST change as recorded in the central Baltic Sea was not local and affected environmental conditions throughout the Baltic. The model provides a causal mechanism between temperature change and environmental conditions as reflected in the sedimentary record (laminated vs. homogenous sequences), and simulations under this model show that during the more unstable and rather cold phase before 6,000 cal. yr BP the hypoxic area (laminated sediments) in the Paleo-Baltic was restricted to the formerly deep northern basin and covered an area of only 20,000 km^2^. After the rapid warming at 6,000 cal. yr BP and during the regional Thermal Optimum the hypoxic area increased (resulting in deposition of laminated sediments) to more than 100,000 km^2^ and spread from the northern basins to the entire Baltic (Fig. 3A; Fig. S4). Therefore the simulation results support a theoretical study^12^ arguing for a positive feedback mechanism between temperature and hypoxia (laminated sediments). Two factors are crucial for this positive feedback loop: i) blooms of N_2_-fixing cyanobacteria which can only develop at summer SSTs >16 °C (refs^13,14^), and ii) phosphate availability. Phosphorus mineralized in sediments under oxic (cold) conditions becomes to a certain extent bound in phosphorus-iron complexes and is retained in the homogenous sediment. A change to hypoxic/anoxic conditions as a result of temperature induced cyanobacteria blooms liberates the iron bound phosphorus and it is released as phosphate to the water column^15,16^. This surplus phosphate in the water column in turn stimulates further cyanobacterial blooms resulting in a substantial increase in primary productivity during warm climatic phases. This additional production of biomass (in the modern central Baltic approximately half of the sedimentary organic carbon is estimated to be derived from these blooms^17^) results in an increased carbon flux into the bottom water and in turn the oxygen demand for heterotrophic processes increases. Bottom-water oxygen concentrations decline and increasingly larger areas become covered by anoxic waters (laminated sediments), resulting in even more phosphate release. Nutrient concentrations increase and as a final result of this positive feedback loop a eutrophic state of the Baltic Sea is established. Our simulations suggest that this state is almost independent of external nutrient loading and can only be changed by reducing summer SSTs (<16 °C), preventing heavy cyanobacterial blooms.

**Figure 4:**
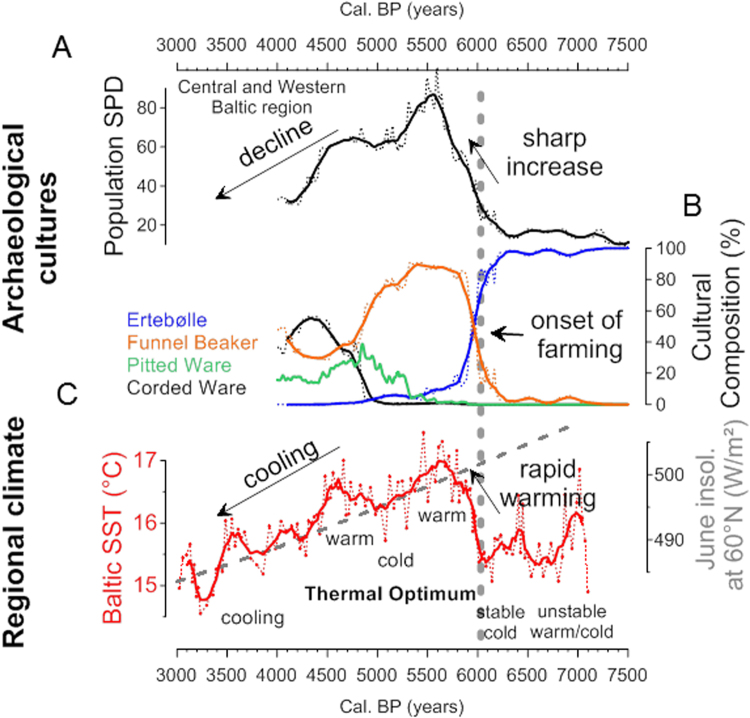
Mid-Holocene SPD records from the Baltic Region in relation to climatic changes. (**A**) Population proxy using a summed probability distribution (SPD) of archaeological radiocarbon dates within the polygon indicated in Fig. 1. Y-axis represents the probability density, on a relative scale. (**B**) Cultural composition (%) based on the subset of the radiocarbon dates with cultural affiliations assigned by the original excavator (58% of full dataset used in (**A**)). Note the rapid change at the boundary between the Meso- and Neolithic, coincides with a substantial increase of the population. Dotted lines show the complete time series, solid lines show a 200 yr rolling mean, to dissuade overinterpretation of small scale wiggles from the calibration curve. (**C**) Summer SST record based on TEX_86_ palaeothermometry. The red line plots the 5-point rolling mean. June insolation at 60°N (in W m^−2^) is plotted as a grey dashed line. The population levels increased synchronously with a period of warming after 6,000 cal. yr BP.

The regional temperature rise causing widespread environmental changes in the entire Baltic region must have also influenced terrestrial settings. Indeed, our Baltic Sea Holocene Thermal Maximum (6,000 to 4,500 cal. yr BP) is also seen in pollen based reconstruction of summer temperature for NW Europe^18^ and Finland^19^. We reconstructed fluctuations in the local human population through time using a Summed Probability Distribution (SPD) of 1,960 terrestrial radiocarbon dates from 608 archaeological sites (Fig. 4A), taken as a proxy for variation in human population levels in the Baltic region through time. The rationale behind this approach is that to a first order of approximation, periods of increased population will leave more dateable material behind in the archaeological record^3,20^ (see SI for further discussion). Evidently, the large climatic and environmental change at 6,000 cal. yr BP that took place in northern Europe is concurrent with the start of a massive increase in population levels. Between 6,000 and 5,600 cal. yr BP the summer climate substantially increased by 2 °C in parallel with a 3.3 fold increase in population levels (Fig. 4). This represents a population growth rate of 6.1% per 25 year generation compared to 1.9% previously between 7,000 and 6,000 cal. yr BP, though these may be slight overestimates given the possible effect of taphonomic loss over time. For comparison, if Surovell’s proposed taphonomic correction formula^21^ is applied, these numbers fall slightly to a 3.0 fold increase, with growth rates of 5.7% and 1.5% per generation respectively. Additionally, the strong relationship between temperature and population is evident throughout the entire overlap in both time-series from 7,000 to 4,000 cal. yr BP (Pearson’s R = 0.73, p-value < 0.00002; see methods). Furthermore, cross-correlation analysis shows the population lagged behind climate by 60 years, suggesting a rapid human response to climate changes.

The mechanism for how the summer temperature increase at 6,000 cal. yr BP could cause a rapid population increase in this particular region can be understood in terms of the improved environmental conditions extending the growing season for domestic crops in a previously marginal area. Nearby regions to the south were already farming, and would have been able to rapidly expand their agrarian economy north as conditions improved. This results in a substantial increase in the available terrestrial biomass per unit area for human consumption, compared to a closed-canopy temperate forest^22^. The availability of more food resources then supports a greater birth rate^23^, which in turn creates a greater demand for and dependency on food production. Our analysis of the radiocarbon record (Fig. 3C) is consistent with this, showing both a substantial population increase and a cultural transition to farming both occurring at 6,000 cal. yr BP.

Evidence of this economic revolution is seen in archaeobotanical and faunal evidence showing the simultaneous introduction of wheat, barley and domestic animals throughout southern Scandinavia ca. 6,000-5,700 cal. yr BP^24^. This transition reflects an important cultural shift, from the Ertebølle culture, representing hunter-gatherer-fisher societies, to the agricultural Funnel Beaker culture (Figs 3C and 4B), though some exploitation of marine resources seem to have continued (see SI). Modeling studies indicate that a warming climate in Scandinavia could induce shifts in the northern limit of cereal suitability by 100–150 km per °C (ref.^25^). Ancient DNA^26,27^ and archaeological evidence^28^ suggests that the transition occurred as farming communities to the south responded to the warming climate by expanding north into southern Scandinavia. By around 5,600 cal. yr BP population levels and summer temperatures reach a maximum (Fig. 4B) and farming is very extensive, with large areas of cleared land^29^.

However, this still leaves the question of whether the improved conditions for farming produced by increasing temperatures at 6,000 cal. yr BP (Fig. 3B) were solely responsible for the observed change from foraging to farming, or whether other factors were at work. In addition to temperature changes, the development of widespread hypoxia in the whole Baltic Sea including coastal areas (Fig. 3A) may also have played a role in the rapid transition from the Ertebølle to the Funnel Beaker culture in the Baltic region, by having a negative effect on the availability of major marine resources. At the beginning of the early Neolithic, ~6,000 cal. yr BP, summer SSTs started to increase rapidly and bottom water conditions in wide areas became persistently hypoxic (Fig. 3A), a state that continued for most of the next two millennia during a local Thermal Optimum (when SST >16 °C, Figs 4 and S4). Hypoxia in the present-day Baltic Sea has been shown to contribute to a reduction in fish populations^19,30^ (see also SI), therefore it is plausible that a development of widespread hypoxia at 6,000 cal. yr BP would have had a similar detrimental impact on fish stocks, particularly the reproductive volume for cod (Fig. S7 and SI). To test this we carried out an analysis of the relative proportions of remains of aquatic and terrestrial fauna from 96 coastal sites, which reveals substantial variation in the aquatic proportion across different site-phases (Fig. S8). A permutation test cannot reject the null hypothesis of no change through time, with the average proportion of aquatic species in the period 6,500 to 6,000 cal. yr BP of 35.6% (95% CI = 26.6% to 44.5%) remaining very similar in the period 6,000 to 5,500 cal. yr BP at 36.6% 995% CI = 22.9% to 50.0%), n = 96, p = 0.941. This analysis, therefore, does not support the hypothesis of an overall reduction in aquatic resource. Further work will be needed in the future to explore this apparent contradiction with the evidently increased hypoxia.

After the peak at 5,500 cal. yr BP, the SPD population proxy shows a steady decline throughout the remainder of the record (Fig. 4A), synchronous with the declining summer insolation at 60°N and summer SST (Fig. 4C), indicating that climatic deterioration played a role in the decline in human population, probably due in part to poorer conditions for farming. At this time there is evidence of woodland regeneration, which would result from a decrease in human activity^29^. This period also sees the southward expansion of the Pitted Ware Culture^26^ (Fig. 4B), during a period when environmental conditions were worsening further north^2^.

Our data reveals that climate change played a key role in demographic and cultural changes leading to the appearance of farming in mid-Holocene southern Scandinavia after a 1,500 yr delay via rapid warming ca. 6,000 cal. yr BP which improved terrestrial conditions for farming. This incited an expansion of the agrarian groups from the south^26^ resulting in the introduction of farming and as a consequence a massive population increase in the Baltic region. Subsequently, the long term cooling from ca. 5,600 cal. yr BP (most pronounced after 4,500 cal. yr BP) drove a gradual decrease in farming productivity causing a reduction in population size and contributed to a later resurgence of hunter-gatherer-fisher communities. Our study suggests a rapid human reaction within only two to three generations to the new opportunities created by the improved conditions for farming, emphasizing the remarkable plasticity humans can demonstrate in response to climate changes.

## Methods

### Sampling and preparation

Sediment core 303600 was collected from the central Baltic Sea in the Gotland Basin just east of Gotland Island at 56°55.02′ N and 19°19.98′ W (Fig. 1, Table 1) using a gravity corer during a cruise of the research vessel (R/V) “Professor Albrecht Penck” in July 2009. The collected core was cut in 1-m sections and split lengthwise. The core halves were line-scanned (photographic documentation) and sampled at a 1 cm resolution. The samples were freeze-dried, ground, and homogenized by mortar and pestle for analysis. The sediment chronology was developed using gravity cores 370540-7 and P435-2-1 taken during cruises with R/V “Aranda” in 2009 and R/V “Poseidon” (P435) in 2012, respectively, in the eastern Gotland Basin. For comparative studies a number of cores were taken during several cruises between 2005 and 2014 in almost all Baltic Sea basins (Fig. S1, Table 1).

### Radiocarbon dating

The sediment cores were radiocarbon dated using picked benthic forminiferal tests (i.e. *Elphidium* spp.), which were present in high numbers in cores P435-2-1 (220 m water depth) and 370540-7 (242 m water depth) from the deepest part of the eastern Gotland Basin as a result of strong saline water inflows^31^. Individually picked benthic foraminifer samples (69 in total) were radiocarbon dated using accelerator mass spectrometry (AMS) to obtain a highly resolved age-depth model. Eight samples of 3-5 mg foraminiferal test were leached to remove any potential surface contamination, before the remaining 70% was decomposed to CO_2_ under vacuum and converted to graphite for AMS ^14^C measurement at the Poznan Radiocarbon Laboratory in Poland. The majority of the samples was too small for routine AMS measurements on graphite and was dated with a new technique at ETH Zürich employing an AMS system equipped with a sample introduction system based on a gas ion source^32^. Foraminiferal tests (0.3-1 mg) were leached with 100 µl of 0.02 M HCl to remove 100 µg carbonate (12 µg carbon), before the remaining carbonate was completely dissolved in phosphoric acid in 3 ml septa sealed vials and the formed CO_2_ was introduced into the ion source of the AMS for ^14^C measurement^33^. The foraminiferal abundance and quality in the lower core interval of 370540-7 is much higher than in the equivalent depth interval of P435-2-1. Therefore, high resolution dating of this older interval concentrated on core 370540-7. Nevertheless, in order to secure a sound and robust high-resolution composite (P435-2-1 and 370540-7) age-depth model, overlapping depths intervals in P435-2-1 were also dated (Figs S2 and S3). The raw AMS ^14^C dates of benthic foraminiferal samples were calibrated using the Marine13 database in the Calib7.01 (ref.^34^) and adjusted with a standard reservoir age of 400 years commonly used in western Baltic Sea studies (Table S1). Since benthic foraminifera only occurred after strong saline water inflows in the saline, oxygen-rich deep water body the use of this reservoir age is deemed adequate.

### Total organic carbon (TOC) content and Loss on ignition (LOI)

The TOC content of sediments of core 303600 and P435-2-1 was determined by measuring the total carbon (TC) using the EA 1110 CHN analyzer (CE Instruments) and the total inorganic carbon (TIC) using the Multi EA- 2000 Elemental Analyzer (Analytic Jena); the TOC content was calculated as the difference between TC and TIC. The LOI was determined by ashing freeze-dried samples at 550 °C for 3 h and calculating the resulting mass difference in wt.%. For Baltic Sea sediments, the LOI provides a proper estimate of the TOC content of the sediments^35^ (see also Fig. S2).

### Correlation of cores

Benthic foraminifera are absent in shallow water sediment cores (like 303600) since incursion of saline waters did not reach this shallower site. Central Baltic Sea cores can be correlated to by comparing loss on ignition (LOI) records, which reflect the burial flux of organic carbon to the sea floor, which is related to primary productivity and bottom water oxygenation. Hence, the age-depth relationship developed on the central Baltic Sea cores from the deeper cores, P435-2-1 and 370540-7 was transferred onto shallower cores from the central/southern Baltic by correlating LOI and/ or organic carbon profiles (Figs S2 and S3) as performed earlier^9,36^. The 370540-7 depth scale was transferred onto P435-2-1 equivalent depth scale by correlating the LOI profiles using Analyseries Software. The resulting relationship for age versus P435-2-1 equivalent depth is shown in Fig. S3. A polynomial fit was applied for the age model and this model was transferred onto 303600 using high-resolution LOI data in Analyseries Software. AMS ^14^C dating of sediment cores from the northern Baltic Sea basins were performed in order to obtain information on the approximate duration of the early Littorina Sea Phase hypoxic stage (Table S1).

### TEX_86_ analysis

Typically 1-3 g of freeze dried sediment were extracted using the Dionex^TM^ accelerated solvent extraction with dichloromethane/methanol (9:1; v/v) as solvent. The total lipid extract was dried over a Na_2_SO_4_ column and then separated using Al_2_O_3_ column chromatography. The polar fraction was obtained using dichloromethane/methanol (1:1. v/v) as the eluent. The polar fraction was concentrated under N_2_ gas and then dissolved in hexane/isopropanol (99:1, v/v) before being filtered using a 0.4 µm PTFE filter as described previously^37^. The sample was then analyzed by high-performance liquid chromatography coupled with atmospheric pressure chemical ionization-mass spectrometry (HPLC/APCI-MS) as described previously^38^. A local TEX_86_ calibration for the Baltic Sea was previously established^9^ using nine surface sediment samples from the Baltic Sea and comparing the TEX_86_ derived SSTs with satellite sea surface temperature (SST) data. The highest correlation was found using the TEX_86_^L^ index^39^ and with the SST of the months July through October. The resultant Baltic Sea summer SST calibration equation is:

$${\rm{SST}}={\rm{34.03}}\times {{{\rm{TEX}}}_{{\rm{86}}}}^{{\rm{L}}}+36.73({{\rm{R}}}^{{\rm{2}}}={\rm{0.89}},\,{\rm{n}}={\rm{9}})$$

### Model Simulation

To simulate the climatic impact on biogeochemistry of the Baltic Sea, we used a coupled three dimensional hydrodynamic and biogeochemical model. The hydrodynamic part of the model is based on the circulation model MOM (version 5.1)^40^ and has been adapted to the Baltic Sea with an open boundary condition to the North Sea, and freshwater riverine input. The MOM model is complemented with a sea ice model to estimate ice cover thickness and extent. Furthermore, a parametric surface wave model^41^ is coupled to MOM with the main purpose of controlling the resuspension of sedimentary material. The biogeochemistry was integrated using the ERGOM biogeochemical model (www.ergom.net), which has been developed for the Baltic Sea, and describes the dynamics of nitrogen, oxygen and phosphorus including the inorganic nutrients nitrate, ammonium and phosphate, particulate organic matter consisting of phytoplankton (autotrophs), dead organic matter (detritus) and zooplankton (heterotrophs). In the ERGOM model, *in-situ* organic matter is produced from the inorganic nutrients by three functional groups of phytoplankton: large cells, small cells and others, and cyanobacteria. Organic material sinks and enters the model sediment as benthic nitrogen and phosphorus. All model processes requiring electron acceptors (e.g. respiration) reduce the oxygen concentration in the water. When oxygen is depleted, nitrogen is first used as an electron acceptor (denitrification) and then subsequently sulfate becomes the electron acceptor resulting in the formation of hydrogen sulfide.

During the Holocene the northern Baltic Sea lifts up and becomes shallower due to the glacio-isostatic rebound, affecting its bathymetry. To reconstruct the Baltic Sea bathymetry about 6,500 cal. yr BP, we used published data^42^ for relative sea level change. These data were combined with a contemporary bathymetry of the Baltic region^43^. The northern basins are sometimes >100 m deeper, while the southern Baltic remains nearly unchanged (Fig. S5). Due to the tremendous computational load (2,000 years have been simulated), we applied a relative coarse model grid of 6 nautical miles (Fig. S5). Vertically the model is resolved into 80 layers. Close to the surface the vertical resolution starts with 0.5 m extending to the bottom to a maximum of 6 m.

Atmospheric forcing was reconstructed using the coastDat data set^44^, a dynamical downscaling re-analysis for the years 1948–2014. For the different climate states (warm vs. cold), we modified the atmospheric data by a delta change approach successfully applied earlier^9^. This approach is typically applied when meteorological data are not available in a sufficient temporal and spatial resolution. In order to simulate the biogeochemical consequences of the change in summer SST that occurred in the reconstructed SST record at 6,000 cal. yr BP, we applied the following conditions. For the warm climate state we increased the 10 m air temperature of the coastDat data by 1 K. To mimic a colder climate we reduced the 10 m air temperature by 2 K and decreased the downwelling shortwave radiation. The effect is a mean SST difference of 2 K between cold and warm climate periods. The temperature difference is higher in summer and amounts up to ca. 3 K (Fig. S6). This temperature change reflects the reconstructed temperature difference for the transition at 6,000 cal. yr BP (Fig. 2).

Pre-industrial nutrient loads to the Baltic Sea due to riverine discharge and atmospheric deposition are derived from previous estimates^45^. We used their estimates for AD 1850 and reduced them by additional 10% to account for a smaller population density at 6,500 cal. yr BP.

We performed the simulations with our model in 50-year time slices, which were eventually concatenated into the complete simulated time-series. The first time slice was initialized using data from a pre-industrial reconstruction of the Baltic Sea’s environment^40^, which is characterized by low nutrient and high oxygen concentrations in the waters of the Baltic. The output of the preceding time slice was used as the starting condition for the next time slice in the simulation. Each time slice was forced by the modified meteorological data producing respective warm or cold climatic conditions. The modeled cold period (350 yr) mimics the cold climate before 6,000 cal. yr BP and the modeled warm phase (400 yr) the warm climate after 6,000 cal. yr BP (Fig. S6).

### Overall population and cultural composition proxies

Based on the premise that periods of increased human population will leave behind more dateable archaeological artifacts^1,13^, local population fluctuations in the Baltic region were reconstructed using a Summed Probability Distribution (SPD) of 1,960 terrestrial radiocarbon dates from 608 archeological sites (see refs^46,47^ for responses to criticisms of this approach; for comparing radiocarbon and other proxies see ref.^48^ ^14^C dates (Fig. 4A black line, top) that fall within the Central and Western Baltic kml polygon (brown polygon Fig. 1) were obtained primarily from the EUROEVOL radiocarbon database (http://discovery.ucl.ac.uk/1469811/). Some additional dates, which became available after publication of the EUROEVOL database were also added. The polygon was specifically constructed to test the hypothesis of a demographic response to climatic change across the broad region of Central and Western Baltic where farming was adopted c.6000 BP. By utilizing the largest possible sample size (site n = 608, sample n = 1960) we can expect local variability (e.g. from endogenous causes) to destructively interfere, and any patterns common to the entire region to be maximized. All these ^14^C dates have an anthropogenic association and are of terrestrial origin from archaeological sites. The SPD was constructed using methods previously described^3,13^. At each site, similar dates (within 200 ^14^C years) were binned, calibrated using Intcal13, summed and normalized to unity, such that multiple similar dates at a site had the same overall weighting in the SPD as a single date. This is a conservative approach to dealing with differential dating at sites due to, for example, a larger research budget permitting more samples to be radiocarbon dated. All binned SPDs were then summed and the final SPD was normalized to unity. The SPD provides a proxy for relative population levels through time within the polygon, but the density values are relative and therefore the units are arbitrary.

We also constructed SPDs from four subsets of ^14^C dates (Fig. 4B) from the Central and Western Baltic polygon, utilizing a total of 1144 of the 1960 ^14^C dates (58%) which had been assigned to one of the following cultures by the original excavator: Ertebølle (or Lihult), TRB (Funnel Beaker), Corded Ware, Pitted Ware. Dates that were considered transitional between two cultures were not included, i.e. Ertebølle/TRB (n = 79) and TRB/Bell Beaker (n = 1). Also not included were 24 dates associated with cultures beyond the temporal interest of this study (for example Early Bronze Age). The remaining 712 excluded dates had not been associated with a specific culture. SPD analysis was performed as described above for all dates in the polygon, with the exception of the final step of normalization to unity. This ensures a fair comparison of the magnitudes of each cultural SPD. These four probability distributions were then summed, and the percentage contribution of each of the four was calculated, thus rescaling each SPD to a percentage between zero and 100.

### Correlation tests between SST and Central and Western SPD

Correlation between SST and Central and Western Baltic SPD was calculated using two methods. In both cases interpolation was required to simplify and match the SPD time series to exactly the same 99 unequal discrete SST time points that overlap with the SPD, from 4,036 to 7,099 yrs BP inclusive. The first method uses Pearson’s product moment correlation coefficient, r = 0.731 (95% CI = 0.624 to 0.812), p-value < 2.2e-16, t = 10.56, df = 97. However, there is the possibility of some spurious shared information between the SST and SPD, which may exaggerate the p-value. For example, SST core time points were estimated using a ^14^C time depth model from dates that were calibrated via the same Intcal13 curve. Similarly both time-series are subject to the same atmospheric variability in ^14^C. Therefore, a second method using a Monte Carlo simulation approach as previously employed^3^ generated 50,000 simulated radiocarbon datasets (using a uniform distribution between 4,000 and 8,000 cal. yr BP). An SPD was generated for each, which were then subjected to exactly the same correlation test with SST. This ensured each simulated SPD was processed using the same calibration and interpolation as the observed radiocarbon dataset and resulted in 50,000 Pearson’s product moment correlation coefficients ranging from −0.735 to + 0.721 (SD = 0.228), which were compared to the observed correlation coefficient. Not a single simulated correlation coefficient was equal or greater (1-tailed) and only one simulation was greater in magnitude but negative (2-tailed), providing a more conservative p-value estimate of 0.00002.

Both the SPD and SST time series were interpolated to annual points between 4,036 and 7,099 yrs BP, allowing a fine resolution cross-correlation analysis using Pearson’s product moment correlation coefficient in the lag range of ± 15% (460 years). A peak correlation was obtained when the SPD lagged SST by 61 years (r = 0.862 compared to r = 0.839 at lag = 0). Note only the relative values are informative as the magnitude of these values is inflated compared to the correlation test since both time series were heavily interpolated and trimmed to ensure a constant window length.

### Analysis of faunal data

Zooarchaeological data from published Mesolithic/Neolithic archaeological sites in the Baltic region were recorded in a relational SQL database. The total Number of Identified Specimens (NISP) for all categories of anthropogenic faunal deposits were recorded, including wild and domestic mammals, birds, fish, molluscs, reptiles, amphibians and crustaceans. Intrusive faunal elements, where identified, were excluded from the database. Taxa were entered at the level of identification published by the original analysts, whether to species or genera, or at the more general level of family (or ‘type’), or according to body size (e.g. large/small mammal). Each taxonomic entry was then associated with a cultural phase e.g. Ertebølle, Pitted Ware etc, and assigned a unique phase identification code. Any published radiocarbon dates derived from the same archaeological contexts of that phase were recorded with the same phase identification code to ensure accurate site phase chronology (see SI). We combined multiple sources of chronological evidence in a Bayesian framework using OxCal, including radiocarbon dates at the target site-phase, date ranges estimated by the excavator using typological evidence, and radiocarbon dates from neighbouring site-phases from the same culture or period (see SI). We used a two-tailed permutation test to fully integrate the uncertainty in the date estimate of each site-phase, and sampling uncertainty in NISP counts (see SI).

### Data availability

Upon publication the datasets generated in the current study will be available in the Pangea repository (www.pangaea.de).

## Electronic supplementary material

Supplementary PDF File
