# Supplementary material for: Climate induced human demographic and cultural change in northern Europe during the mid-Holocene
Source: Sci Rep. 2017 Nov 10;7:15251. doi: 10.1038/s41598-017-14353-5 (PMC5681586; doi:10.1038/s41598-017-14353-5)
Supplement: Supplementary file 1 — Supplementary PDF File [file 41598_2017_14353_MOESM1_ESM.pdf]

# – SUPPLEMENTARY INFORMATION –

Climate induced human demographic and cultural change in northern Europe during the mid-Holocene

L. Warden, M. Moros, T. Neumann, S. Shennan, A. Timpson, K. Manning, M. Sollai, L. Wacker, K. Perner, K. Häusler, T. Leipe, L. Zillén, A. Kotilainen, E. Jansen, R.R. Schneider, R. Oeberst, H. Arz, and J.S. Sinninghe Damsté

1. Human population and cultures in the Baltic area

Although there is now evidence of the presence of domestic pigs in Ertebølle Mesolithic contexts in Baltic north Germany ca. 6,500 cal. BP[1], the arrival of farming in southern Scandinavia is associated in material culture terms with the Funnel Beaker, or TRB culture, beginning ca. 6,000 cal. BP. Ancient DNA evidence has recently shown that it involved an expansion of farming populations from further south[2-3]. In terms of the economy, farming is indicated by the presence of cereal remains and the bones of domestic animals. Direct radiocarbon dates on charred cereal grains of wheat and barley show their presence across southern Scandinavia as far north as middle Sweden in the period 6,000-5,700 cal. yr BP and the same is true for directly dated domestic animal bones, including sheep/goat and cattle[4]. However, this does not mean that wild resources ceased to be exploited. When coastal and lakeside sites are distinguished from inland ones they generally show very high proportions of wild fauna and even at inland sites wild fauna can represent a significant proportion at sites from the EN I phase[4], 6,000-5,500 cal. yr BP, although there is a shift towards higher domestic proportions in later phases at all sites[4]. It remains unresolved whether local forager groups continued to exist in coastal zones side by side with incoming farmers or whether the latter exploited marine resources on a seasonal basis. There is evidence of some introgression of genes from hunter-gatherer lineages of the 'Western Hunter Gatherer' group into the farming population but this could have happened to the ancestors of the TRB population during the Middle Neolithic in Central Europe[2A]; as yet no introgression has been found in the opposite direction[2].

In recent years extensive stable isotope and lipid analyses have been carried out on both human bone and ceramic residues to examine dietary patterns during the Mesolithic and Neolithic of southern Scandinavia. A stable isotope study of human bone[5] indicated a wide-ranging diet for Mesolithic individuals, including partially terrestrially-based protein. Neolithic diets, on the other hand, were much more uniform and based entirely on terrestrial protein, most probably from domesticated resources. Fischer et al.'s isotope study[6] of human and dog bones from Mesolithic and Neolithic sites found that Mesolithic samples indicated heavy dependence on marine resources, especially high trophic level fish, even at inland sites. With one exception, the Neolithic samples indicated a general shift towards a diet based on terrestrial foods with some low trophic level freshwater and marine food. However, most of these samples come from the Early Neolithic II period and later; i.e. after 5,500 cal. yr BP. Very few early Neolithic samples from coastal sites have yet been analysed[4].

Isotope analyses of ceramic residues[7] from late Mesolithic Ertebølle pottery from the coastal site of Tybrind Vig in Denmark indicated that the vessels were mainly used 'for processing marine products', and that marine products were strongly represented at the inland site of Ringklosters. The results of analyses of residues from TRB vessels indicated the processing of freshwater fish as well as terrestrial products including animal fats. A further study[8] of a much larger number of ceramic residues from Ertebølle and early Neolithic TRB (6,000-5,500 cal. yr BP) vessels in Denmark and the Baltic coastal area of Germany found that EN vessels from coastal sites had been heavily used for marine animals while evidence of freshwater resources was found at inland sites. However, a third of the TRB vessels, including samples from the coastal sites, also had evidence of dairy fats indicating the keeping of animals for milking. It seems then that there was not a complete switch to domesticated resources with the local beginning of farming and that, whoever the farmers were, they initially combined both marine and domestic animal resources. Analyses of TRB EN ceramic residues from the site of Skogsmossen in eastern middle Sweden[9] produced similar results to those of Craig et al.[8], including evidence of both dairy fats and marine resources.

Isotopic analyses of human bones from the island of Oland in the Baltic show a contrast between the MN TRB site of Resmo, ca. 5,400-4,800 cal. yr BP, which has the pattern described above showing a mixture of marine and terrestrial resources, and the contemporary Pitted Ware Culture (PWC) site of Kopingvik, which shows a heavy reliance on marine mammals with a possible addition of marine fish, as in the Mesolithic phase at the site[10]. This pattern is confirmed by results from the eastern Swedish coastal PWC site of Korsnas, dating to ca. 5,300-4,800 cal. yr BP, where the proportion of terrestrial protein in the diet was negligible and virtually all individuals analyzed had diets indicating seal consumption, with the outlier pointing to a diet of fish as well as seal[11]. The faunal assemblages from Pitted Ware sites on the island of Gotland (see ref. 12, Table 15.1, p. 291) support this picture. Domestic animals are either completely or virtually absent, there are numbers of wild boar, and very large numbers of seals and fish. Further north, recent lipid analyses of ceramic residues from the Combed Ware site of Vantaa Stenkulla/Maarinkunnas in southern Finland ca. 5,900-5,300 cal. yr BP, thus contemporary with the TRB further south, show that they derive from resources of marine origin, in keeping with the archaeological remains from the site[13].

The long-standing suggestion that the southward spread of the Pitted Ware to Denmark represents a re-expansion of hunter-gatherer populations from the north has been supported by a genomic analysis of ancient DNA samples from Pitted Ware skeletons from Gotland[2,14]. This showed continuity from a late Mesolithic to the PWC samples, and that all belonged to a group of Mesolithic populations that was very widely distributed across Europe. The difference in their genomes between the PWC samples and that from a TRB farmer burial in central southern Sweden was greater than that between the two present-day European populations that are most different from one another, Finns and Italians.

Finally, the Single Grave/Battle Axe/Corded Ware culture (all different regional names for essentially the same set of material culture) appears in southern Scandinavia and the Baltic area from ca. 4,800 cal. yr BP and succeeds the TRB, running alongside the later part of the PWC. Like the TRB this is an agricultural culture, though with probably more emphasis than the latter on domestic animal exploitation. Like the latter, it represents at least in part an expansion of new populations into southern Scandinavia[3,15]. Recent stable isotope and lipid work on ceramic residues from Corded Ware sites in southern Finland[13] dated *ca.* 4,500 cal. yr BP has produced patterns corresponding to terrestrial ruminants at 60° north even though the sites were close to the coast, and half of the patterns correspond to dairy fats indicative of milking, although a single vessel had evidence of marine product processing. In contrast, the subsequent Kiukainen culture, with pottery similar to both Corded Ware and local Late Comb Ware, had residues indicative of processing both marine and terrestrial ruminant products in the same vessels, thus a shift to use of both domestic and marine resources, after 4,500 cal. yr BP.

2. Impact of hypoxia on marine resources in the Baltic Sea

In this section we discuss in detail the impact that the basin-wide development of hypoxia at around 6,000 cal. yr BP and associated temperature changes might have had on the maritime resources in the Baltic Sea. We start with describing the effect of spreading hypoxia on fish stocks (i.e. cod and herring) in the recent Baltic Sea. The marked spread of hypoxia during the last century represents, however, only a somewhat limited analogue to the tremendous change in environmental conditions we observe in our palaeorecord at 6,000 cal yr BP. We also discuss the observed (published earlier) changes in Danish kitchen midden composition at 6,000 cal. yr BP.

2.1 Impact of warming and spread of hypoxia on the Baltic cod (*Gadus morhua*) population in the recent Baltic Sea.

During the last decades, extensive laboratory and field studies, supported by available long-time instrumental hydrographic datasets, have focused on the impact of recent climatic and environmental changes on fish stocks. Comprehensive reviews for the Baltic Sea[16-18] clearly show that the fish population in the Baltic Sea has been substantially impacted by recent climatic (warming) and environmental (spread of hypoxia in the Baltic) change that affects fish growth, survival and reproduction rates.
A spread of hypoxia in the deep basins has been observed from the cold Little Ice Age to the Modern Warm Period based on instrumental and sediment proxy data[19-20]. Conley et al.[21] reported an alarming trend of hypoxia in the coastal zone of the Baltic Sea since 1950. The trend is attributed to increased nutrient load but also to the strengthening of stratification due to warming. Hypoxia (oxygen concentration < 2 ml/l) occurs with an episodic character and is most prominent in the Danish Straits, and in the Swedish and Finnish archipelagos. Hypoxia impacts bottom-living organisms and impairs fish habitats (see below).
Climate and associated environmental changes can affect fish populations by *direct* and *indirec*t effects. Direct effects include physiological effects on growth and maturation, behavioural effects that alter migration and distribution, and displacement effects brought about by alteration of circulation patterns that transport and disperse eggs and larvae. Indirect effects include changes in the seasonal production of planktonic crustaceans, especially copepods, which form the larval diet of most fish species, and other complex food-web effects that result from changes in prey and predator communities. Temporal overlap of peak larval and prey abundance is critical.
Cod (*Gadus morhua*), herring, and sprat are by far the most important fish species in the present-day Baltic[22] and likely also during the Holocene when the Baltic was a brackish basin. Cod is considered the top predator in the present-day Baltic, predominantly feeding on adult herring and prat, but earlier in the Holocene cod was an important prey for harp seals. Here we briefly discuss direct and indirect effects of changing hydrographic conditions on the stock of these fish.

Direct effect of hypoxia on cod recruitment. Eggs of the Baltic cod successfully develop only in deep waters with oxygen concentrations > 2 ml/l (e.g., refs. 23-24) and a salinity >11 psu (ref. 25). These thresholds mark the so-called reproductive volume, i.e. the water volume sustaining cod egg development[24]. After spawning, cod eggs sink to a depth at which they are neutrally buoyant and float, in waters with a salinity of about 11 psu; conditions often occurring at the halocline in the deeper basins. If water at this depth is too low in oxygen (i.e. during stagnation periods), the eggs die since there is no egg survival under hypoxic conditions[23]. The climatically-controlled spread and consequent decrease in reproductive water volume during stagnation periods since 1980s caused high cod egg mortality in the Gdansk and Gotland Basin[19]. The reproductive volume is also influenced by surface water temperature as cod spawns in a narrow rather cold temperature range of 5-8°C (e.g., refs. 26-27). Egg mortality increases rapidly at temperatures >10°C (e.g., ref. 23).

Indirect effect on fish stocks - influences of mesozooplankton on larval survival. The co-occurrence (degree of temporal overlap) of peak prey and larval abundance is critical for high survival rates. Higher temperatures, low oxygen conditions and salinity lead to a decline in the stock of "cold water" *Pseudocalanus* sp., the main pray of cod larvae, resulting in low survival rates[16,18,28,29]. In addition, herring growth rates also depend explicitly on the

abundance of the copepod *Pseudocalanus*. As herring grows, their dietary preference change and large nektobenthic species become common prey. The abundance of this latter large prey decrease during hypoxia and large herring are forced to consume small zooplankton which increases foraging costs and leads to a reduced growth in herring as observed during the 1990s and early 2000s (e.g., ref. 30).

Direct effect of oxygen concentration on growth, activity, survival and distribution of fish. Hypoxia also directly impacts the growth, activity, survival and distribution of *Gadus morhua*[31] because oxygen is an ultimate requirement for metabolism which converts food material and available reserves into energy needed for reproduction and activities. The limiting effects of dissolved oxygen to acquire energy for growth and activity can be described by metabolic scope framework[31] and Fry's concept of aerobic metabolic scope[32]. Activity ceases when dissolved oxygen is ≤ 20 % and growth and food ingestion are already significantly reduced below 70 % saturation. The recent increase of the area in the Baltic Sea with hypoxic conditions has resulted in a change of the spatial distribution of Baltic cod and has reduced habitat size[19,33] with risk for fish density-dependence effects.

Direct effect of surface water warming. Warming of the Baltic with a reduction in sea ice cover results in an earlier onset of stratification and spring phytoplankton bloom. Intensified stratification due to less winter time deep mixing causes less vertical mixing of nutrients into the photic zone during the post bloom period. The result is a temporal mismatch between peak prey and peak larval abundance. It has been shown that surface water temperature dominates, beside dissolved oxygen, behavior and ectotherm physiology[34-35], whereas dissolved oxygen is the primary limiting factor and temperature is the control factor[36]. Therefore, the increase of water temperature in the surface layer in summer resulted in a further decrease of the area with optimum conditions to accumulate energy reserves by cod after spawning in summer. The increase of water temperature in the Baltic Sea (e.g. ref. 37) specially in spring and summer, influences the reproduction success of spring spawning herring that spawns shallow waters of the Baltic Sea or in lagoons like Greifswalder Bodden[38,39] or Gulf of Riga[40]. One bottleneck of the reproduction success is the beginning of active feeding by hatch larvae. Copepode nauplii are the first prey type of herring larvae[41-43]. The beginning of reproduction of copepods starts some days later[44], which is determined by time when light intensity exceeds 100 W m^{-2} (ref. 45) and independent of the water temperature. Increasing water temperature in winter and spring will not results in earlier start of phytoplankton and zooplankton reproduction, however, the probability is high that critical water temperature for the spawning of herring (16 - 17°C; ref. 41) and the normal development of eggs (14 – 15 °C; refs. 46-47) are earlier reached resulting in shortened period for optimum reproduction success of spring spawning herring. This is another example for the "match-mismatch" hypothesis of lavea and their prey[48]. North Sea cod stocks occupy areas with water temperature between 1 °C and 11 °C, although total thermal range varies between -1 and 19 °C (ref. 49). A similar temperature range was determined by means of electronic tags (-1.5 °C – 19 °C) with narrower range during spawning season (1 °C – 8 °C)[50]. The optimal temperature for growth of adult cod is 7 °C. Cheung et al.[51] estimated the median, 25 percentile and 75 percentile of temperature preference of *Gadus morhua* with 6, 3, and 9 °C, respectively. Cod is a highly mobile fish and easily migrates to areas with optimal temperature conditions.

In summary, it is clear that warming and the development of hypoxia in the present day Baltic Sea has a negative effect on its fish stocks.

2.2 The present-day distribution and food sources of Harp seal

Harp seal (*Phoca groenlandica* Erxleben 1977) is an Arctic deep-sea species breeding on pack ice (sea-ice) not attached to land in three major areas: the White Sea, around the Jan Mayen Islands, and Newfoundland[52]. The harp seal is not present in the modern Baltic. Its arrival in the Baltic during the Stone Age has been a matter of a long lasting discussion (see below). About two-thirds of the food of harp seals is fish and one third consists of invertebrates[52-53]. Capelin *(Mallotus villosus)* is the most important fish taken in the subarctic, and polar cod *(Boreogadus saida)* the most important fish in the Arctic[52,54]. Polar cod and cod form a significant part of fish prey during times when the capelin stock is low[53]. Hence, when harp seals occurred in the Baltic during the Holocene it is likely that they also foraged on cod.

2.3 Favourable environmental conditions for marine resources in the Baltic Sea during the Mesolithic/ERB

Fish. Our simulation results show optimal environmental conditions for cod and herring growth and recruitment during the Mesolithic just after the major Littorina transgression (Fig. S7) when climate was relatively cold (Fig. 2) and hypoxic conditions were restricted to the formally much deeper basins in the North (Figs. S4 and S7). This suggests the reproductive volume for cod was huge and conditions were favourable. The salinity during this period and also during the transition toward the Neolithic was relatively high. There was likely no hypoxia in coastal waters in summer and the water column was fully mixed during winter time.

Fishing was, indeed, particularly important in Danish coastal waters during the Mesolithic Stone Age (e.g. Enghoff et al.[55], who analysed 100,800 fish bones from settlements). One of the most frequently caught species in the Danish Stone Age sea was Atlantic cod. This study includes Bornholm Island located at the border from the western to the central Baltic. The adjacent Bornholm Basin is one of the recent spawning grounds for Baltic Cod. It should be noted that the main fishing gear used by Ertebølle fishermen was probably stationary self-fishing traps deployed in shallow nearshore waters during summer[56]. Thus, the fish community represented by the archaeological material[55] is a good indicator of the fish community of coastal Danish waters. Also in Northern Germany cod was one of the most caught fish species during the Mesolithic[66].

Harp seal. Based on archeological records, it is concluded that the harp seal was present the Baltic Sea from 7,000 cal. yr BP[57-59] and entered the northern Baltic ca. 6,400 cal. yr BP[60]. Bennike et al.[61] report two peak occurrences of harp seal in Danish waters with the first occurring during the ERT to early TRB time (6,100 to 5,800 cal. yr BP). Osteometric data indicate that these individuals reached the same adult size as extant harp seals of the northeastern Atlantic Ocean populations, suggesting that the seal population during the Mesolithic was closely related to the present Atlantic populations[59]. Since there are no reports of fossil bones of capelin in the Baltic, the main prey of harp seal was most likely cod, which was abundant in the Baltic during this cold phase. A harp seal breeding colony is reported from Northern Germany[61].

Implications for human communities. The appearance of harp seal and the wide distribution of cod seemingly occurred during the cold phase from 6.5 to 6.0 ka BP (Fig. 2) that followed shortly after the marine Littorina transgression. Archaeological data provide evidence that aquatic resources have been an important part of the human diet during this period[55,61-70]. Shell midden accumulation was high and oyster (*Ostrea edulis*) shells occur frequently. This

is also indicative of high surface water productivity[71-72] during this period and is contrary to the present situation with no naturally occurring oysters in Danish waters[69].

2.4 Unfavorable conditions for some aquatic resources developed after the transition from Mesolithic ERB to the Neolithic TRB culture

Fish. At the Mesolithic/Neolithic transition at 6,000 cal. yr BP we observe a sharp temperature increase and a spread of hypoxia (Fig. 4b). The reproductive volume (warm surface layer thickened, stable hypoxic waters are found in wide areas) for hypoxia and temperature-affected fish species appears to have decreased and during summer hypoxic conditions temporarily occurred in coastal areas. As mentioned earlier salinity was not a limiting factor for the reproductive volume at that time. The number of fish bones in Danish kitchen midden reduced at certain sites from the Mesolithic to the Neolithic[73].

Harp seal. Only a very few harp seal finds in western and southern areas are dated to TRB[61]. The same applies to central and northern Baltic Sea areas, where a number of harp seal finds in refuse fauna are dated to ERB and PWC time[58,60,74]; there are almost no dates of TRB age. Two factors can be related to the decline of seals during this period. Firstly, important prey fish stocks were likely significantly reduced after 6,000 cal. yr BP and, therefore, seals were deprived of their main food source. Secondly, a significant decrease in size of adult harp seal is reported from 5,800 cal. yr BP[59]. The ecological rule of Bergmann predicts that evolution favors a large body size in endothermic animals in a cold environment, due to the lower heat loss in these individuals. After 6,000 cal. yr BP the harp seals were exposed to an unusually warm climate for a long period of the year. As a large body size is costly for the individual to reach, and to maintain, the mean body size of the population gradually decreased when a large body was no longer critical[59].

Implication for human communities. Our environmental reconstruction shows extensive warming and spread of hypoxia at 6.0 ka BP (Fig. 4), suggesting the possibility of a substantial decline in the affected aquatic resources at this time. Shell midden accumulation also declined sharply at this time and oysters shells disappeared and were substituted by cockel and mussels remains, which is an indication of lower productivity[69,75]. However, our faunal analysis does not support the hypothesis that total aquatic resources declined, and we fail to reject the hypothesis of a significant change in the aquatic proportion of all NISP through time (Fig. S8).

3. Archaeological data analysis

3.1 Results

To test if the effects of increased hypoxic conditions in the Baltic Sea are seen in archaeological remains, we analysed the Number of Identified Specimens (NISP) of hypoxia/temperature sensitive aquatic species from 81 coastal sites from the entire Baltic area, over the period 8,100-3,900 cal. yr BP. The proportion of hypoxia-influenced aquatic fauna against aquatic fauna overall was used since absolute NISP counts conflate various biases such as differential taphonomy and excavation size. We utilised all coastal site-phases in the database with a minimum 10 NISP and a chronological estimate (n=81). This included a handful of sites outside the demographic polygon (Fig. 1), since we were testing for a Baltic-wide occurrence of hypoxia. The results (Fig. S9) show substantial variation in the proportion of affected species across different site-phases. A permutation test shows a reduction in the proportion of affected species from 17.6% (95% CI = 9.7% to 26.0%) in the period 6,500-

6,000 BP to only 8.4% (95% CI = 2.7% to 16.8%). The p-value of 0.404 suggests we cannot reject the null hypothesis of no change through time, nevertheless this result is consistent with a reduction in the availability of hypoxia and temperature sensitive aquatic species after 6,000 cal. yr BP.

In order to see whether there was a change through time in the use of marine resources overall we used NISP counts to calculate the relative proportions of aquatic and terrestrial fauna. As with the hypoxia /temperature analysis, we utilised coastal site-phases in the database, but constrained these to sites within our demographic polygon (Fig. 1), since this is the area where agriculture was introduced at ~6000 BP and we are interested in the possible impact of hypoxia in producing this transition as a result of decreased availability of aquatic resources. We applied a minimum inclusion criterion of 10 total NISP (rather than only aquatic in the hypoxia /temperature analysis), which increases the number of site-phases to n=96. The results (Fig. S8) show substantial variation in the aquatic proportion of all NISP across different site-phases. The permutation test cannot reject the null hypothesis of no change through time, with the average proportion of aquatic species in the period 6,500 to 6,000 cal. yr BP of 35.6% (95% CI = 26.6% to 44.5%) remaining very similar in the period 6,000 to 5, 500 cal. yr BP at 36.6% 995% CI = 22.9% to 50.0%), n = 96, p=0.941. Therefore, the faunal evidence is consistent with an increase in temperature and hypoxia after 6,000 cal. yr BP, but does not support the hypothesis of an overall reduction in aquatic resources.

3.2 Extended methods

Site-phase chronology. Accurate dating of each site-phase presents a substantial challenge when attempting to identify changes at a temporal resolution of centuries or shorter. Approximately a third of site-phases have zero or one associated radiocarbon date, and only a third have five or more dates. Therefore, we combined multiple sources of chronological evidence in a Bayesian framework using OxCal 4.3 online, which is based on the work of Bronk Ramsey[76] and Reimer et al.[77]. We modelled the intensity of occupation through time as a Gaussian distribution, and used the 'Sigma_Boundary' function to estimate the 1-sigma 'boundaries' of each site-phase. Site reports from 16 phases estimate a start and end date which represents the belief of the expert excavator given a variety of evidence. We used these as priors in their respective OxCal models, after 'converting' this uniform distribution to a Gaussian using the mean and SD of the uniform distribution. For each OxCal model we simultaneously estimated the sigma boundaries of three temporal 'entities': i) The site-phase (informed by radiocarbon dates assigned only to that site-phase); ii) The 'local culture and period range' (informed by radiocarbon dates from all sites within 50 km that have been assigned both the same culture and the same period as the target site-phase); iii) The 'local culture range' (informed by radiocarbon dates from all sites with 50 km with the same culture); and iv) The 'local period range' (informed by radiocarbon dates from all sites within 50km with the same period).

The OxCal model constrains the boundaries of each temporal entity such that the site-phase must occur within the boundaries of the other three temporal entities, as shown in the Venn diagram below. Specifically, the start and end boundaries (S1 and E1) of the site-phase (1) must fall within the boundaries of the 'local culture and period range' (2) since radiocarbon dates from the former must be a subset of the latter; whilst (2) must also be a subset of both the 'local culture range' (3) and the 'local period range' (4).

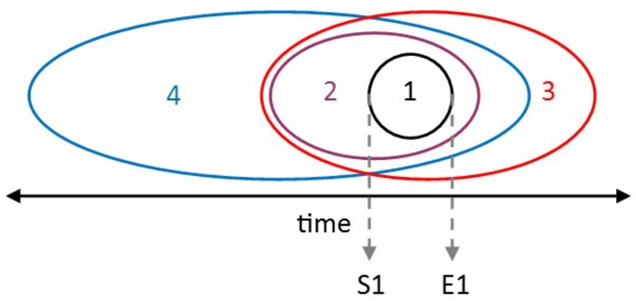

In this way, radiocarbon dates from local sites can improve our estimates of the target site-phase, by incorporating information from site-phases from common cultures and periods. Finally, the posterior distributions of S1 and E1 are summarised as a single point statistic using the median value.

NISP proportions. Absolute NISP counts conflate various biases such as differential taphonomy and excavation size. Therefore we only considered proportions at each site-phase, which we then assumed are equally representative of faunal exploitation of the Baltic. However, larger sample sizes will provide a more accurate estimate of the true proportion. Therefore when estimating the proportion of aquatic species that are influenced by hypoxia and temperature we used the raw NISP counts of those influenced and those not influenced as shape parameters in a beta distribution (plus one count each to represent an equal prior), to estimate a posterior probability distribution of the true proportion. This posterior is used both to plot the aquatic proportions (using the 95% quantiles) and in the permutation test by randomly sampling from it.

Significance tests. We used a two-tailed permutation test to look for a difference between the periods 6,500-6,000 cal. yr BP and 6,000-5,500 cal. yr BP with regard to both a change in the proportion of species influenced by hypoxia and temperature, and a change in the proportion of aquatic species. The test calculates the observed mean proportion for each period, then deducts one from the other. This test statistic is then compared to a null distribution of the same statistic, generated by pooling the site-phases from both periods, then randomly assigning n_1 and n_2 site-phases to each period (where n_x are the observed number of site-phases in each period). However, the test also accounts for the date uncertainty of each site-phase since the wide Gaussian distributions create uncertainty about which period a site-phase belongs to. Therefore for each permutation, a random date is first sampled from each site-phase's Gaussian date range. Using this random date, each site-phase can then be assigned to one or other (or neither) period. Similarly, since the true NISP proportions are uncertain, a random proportion is sampled from each beta distribution. The observed summary statistic is then calculated from these sampled values, and a null statistic is also calculated by pooling and random assignment. The single observed and single null statistic are then compared, and counted if the observed is more extreme. This is repeated for 100,000 permutations, and the total count of 'more extreme' comparisons is summed and divided by 100,000 which provides a p-value estimate. Repeating the entire process confirms the accuracy of this p-value to approximately 0.001.

Confidence intervals. In Figs. S8 and S9 the grey areas indicating the 95%, 75% and 50% CI represent confidence intervals around a rolling mean estimate. Given the substantial variation in NISP percentages across contemporaneous site-phases (there is broad bimodality towards zero and one), we should expect many individual site-phases to site well outside these intervals. The challenge is to fairly incorporate three sources of uncertainty: the error in the date estimate of each site-phase; the error in the percentage NISP of each site-phase; and the

sampling error during periods where only a few site-phases are present. These first two sources are handled using a similar resampling technique used in the permutation test. The time series is arbitrarily divided into 100 year bins, and for each bin the mean NISP proportion is estimated as follows. Firstly, a random date is assigned to each site-phase by sampling from their respective Gaussian distributions. This allows every site-phase to be assigned to one of the bins. Similarly each site-phase is randomly assigned a NISP proportion by sampling their respective beta distributions. So far, this accounts for uncertainty in the chronology and NISP proportions at each site-phase. Sampling uncertainty from each bin having only a small number of site-phases assigned to it is then dealt with by assigning one further 'ghost' site-phase to each bin, with a random NISP proportion sampled from a uniform distribution between 0 and 1. A bootstrapped mean estimate is then calculated for each bin (sampling with replacement all site-phases in each bin). This additional random ghost site-phase has the effect of incorporating a uniform prior. Bins with a larger number of site-phases overwhelm this prior, and the estimated mean becomes closer to the sample mean. For example, in Fig. S8 the large number of Trichterbecher site-phases c. 5,000 cal. yr BP pull the CI towards zero, successfully overwhelming the uniform prior, whilst in contrast the sparsity of site-phases between 4,500-4,000 cal. yr BP are unable to overwhelm the prior, so the CI remains wide. Finally, the entire procedure is repeated 10,000 times, and quantile confidence intervals are estimated for each bin independently.

References

1. Krause-Kyora, B. *et al.* Use of domesticated pigs by Mesolithic hunter-gatherers in northwestern Europe. *Nat. Commun.* **4,** 2348 (2013).
2. Skoglund, P. *et al.* Genomic diversity and admixture differs for Stone-Age Scandinavian foragers and farmers. *Science* **344,** 747–750 (2014).
3. Mittnik, A. *et al.* The genetic history of northern Europe. bioRxiv 113241 (2017); doi: https://doi.org/10.1101/113241.
4. Sørensen L, & Karg S. The expansion of agrarian societies towards the north a new evidence for agriculture during the Mesolithic/Neolithic transition in Southern Scandinavia. *J. Archaeol. Sci.* **51,** 98-114 (2014).
5. Richards, M. P., Price, T. D. & Koch, E. Mesolithic and Neolithic Subsistence in Denmark: New Stable Isotope Data. *Curr. Anthropol.* **44,** 288–295 (2003).
6. Fischer, A. *et al.* Coast–inland mobility and diet in the Danish Mesolithic and Neolithic: Evidence from stable isotope values of humans and dogs. *J. Archaeol. Sci.* **34,** 2125–2150 (2007).
7. Craig, O. E. *et al.* Molecular and isotopic demonstration of the processing of aquatic products in northern European prehistoric pottery. *Archaeometry* **49,** 135–152 (2007).
8. Craig, O. E. *et al.* Ancient lipids reveal continuity in culinary practices across the transition to agriculture in Northern Europe. *Proc. Natl. Acad. Sci.* **108,** 17910–17915 (2011).
9. Isaksson, S. & Hallgren, F. Lipid residue analyses of Early Neolithic funnel-beaker pottery from Skogsmossen, eastern Central Sweden, and the earliest evidence of dairying in Sweden. *J. Archaeol. Sci.* **39,** 3600–3609 (2012).
10. Eriksson, G. *et al.* Same island, different diet: Cultural evolution of food practice on Öland, Sweden, from the Mesolithic to the Roman Period. *J. Anthropol. Archaeol.* **27,** 520–543 (2008).
11. Fornander, E., Eriksson, G. & Lidén, K. Wild at heart: Approaching Pitted Ware identity, economy and cosmology through stable isotopes in skeletal material from the Neolithic site Korsnäs in Eastern Central Sweden. *J. Anthropol. Archaeol.* **27,** 281–297 (2008).
12. Rowley-Conwy, P. North of the frontier: Early domestic animals in Northern Europe. *Orig. Spread Domest. Anim. Southwest Asia Eur.* 283–312 (2013).
13. Cramp, L. J. E. *et al.* Neolithic dairy farming at the extreme of agriculture in northern Europe. *Proc. Roy. Soc. Lond. B Biol. Sci.* **281,** 20140819 (2014).
14. Skoglund, P. *et al.* Origins and genetic legacy of Neolithic farmers and hunter-gatherers in Europe. *Science* **336,** 466–469 (2012).
15. Haak, W. *et al.* Massive migration from the steppe was a source for Indo-European languages in Europe. *Nature* **522,** 207-211 (2015).

16. Möllmann, C., Kornilovs, G., Fetter, M., & Köster, F. W. Climate, zooplankton, and pelagic fish growth in the central Baltic Sea. *ICES J. Mar. Sci.: J. Conseil* **62**, 1270-1280 (2005).
17. MacKenzie, B. R., Gislason, H., Möllmann, C. & Köster, F. W. Impact of 21st century climate change on the Baltic Sea fish community and fisheries. *Global Change Biol.* **13**, 1348-1367 (2007).
18. Köster, F. W., Vinther, M., MacKenzie, B. R., Eero, M. & Plikshs, M. Environmental effects on recruitment and implications for biological reference points of eastern Baltic cod (*Gadus morhua*). *J. Northwest Atlant. Fish. Sci.* **41**, 205-220 (2009).
19. Jonsson, P., Carman, R. & Wulff, F. Laminated sediments in the Baltic: A tool for evaluating nutrient mass balances. *Ambio* **19**, 152-158 (1990).
20. Kabel, K., Moros, M., Porsche, C., Neumann, T., Adolphi, F., Andersen, T. J., Sinninghe Damsté, J. S. Impact of climate change on the Baltic Sea ecosystem over the past 1,000 years. *Nature Climate Change* **2**, 871-874 (2012).
21. Conley, D. J. *et al.* Hypoxia is increasing in the coastal zone of the Baltic Sea. *Environment. Sci. Technol.* **45**, 6777-6783 (2011).
22. Köster, F. W. et al. Developing Baltic cod recruitment models. I. Resolving spatial and temporal dynamics of spawning stock and recruitment for cod, herring, and sprat. *Can. J. Fish. Aquat. Sci.* **58**, 1516-1533 (2001).
23. Wieland, K., Waller, U, & Schnack, D. (1994). Development of Baltic cod at different levels of temperature and oxygen content. *Dana* 10, 16 pp.
24. MacKenzie, B., Hinrichsen, H. H., Plikshs, M., Wieland, K. & Zezera, A. S. Quantifying environmental heterogeneity: habitat size necessary for successful development of cod *Gadus morhua* eggs in the Baltic Sea. *Mar. Ecol. Progr. Ser.* **193**, 143-156 (2000).
25. Westin, L. & Nissling, A. Effect of salinity in spermatozoa mobility, percentage of fertilized egg development pf Baltic cod (*Gadus morhua*) and implications for cod stock fluctuations in the Baltic. *Mar. Biol*. **108**, 5-9 (1991).
26. Hüssy, K. Review of western Baltic cod (*Gadus morhua*) recruitment dynamics. *ICES J. Mar. Sci.: J. Conseil* **68**, 1459-1471 (2011).
27. Brander, K. M. et al. Environmental Impacts - Marine Ecosystems. In *North Sea Region Climate Change Assessment* (pp. 241-274). Springer International Publishing (2016).
28. Hinrichsen, H. H., Möllmann, C., Voss, R., Köster, F. W. & Kornilovs, G. Biophysical modeling of larval Baltic cod (*Gadus morhua*) growth and survival. *Can. J. Fish. Aquat. Sci.* **59**, 1858-1873 (2002).
29. Möller, K. O. *et al.* Effects of climate-induced habitat changes on a key zooplankton species. *J. Plankt. Res.* **7**, 530-541 (2015).
30. Rönkkönen, S., Ojaveer, E., Raid, T., & Viitasalo, M. Long-term changes in Baltic herring (*Clupea harengus membras*) growth in the Gulf of Finland. *Can. J. Fish. Aquat. Sci.* **61**, 219-229 (2004).
31. Chabot, D. & Claureaux, G. Environmental hypoxia as a metabolic constraint on fish: The case of Atlantic cod, *Gadus morhua*. *Mar. Poll. Bull.* **57**, 287–294 (2008).
32. Claireaux, G. & Chabot, D. Responses by fishes to environmental hypoxia: integration through Fry's concept of aerobic metabolic scope. *J. Fish Biol*. **88**, 232-251 (2016).
33. Plante, S., Chabot, D., & Dutil, J.D. Hypoxia tolerance in Atlantic cod. *J. Fish Biol.* **53**, 1342-1356 (1998).
34. Magnuson, J. J., Crowder, L. B. & Medvick, P.A. Temperature as an ecological resource. *Amer. Zool.* **19**, 331–343 (1979).
35. Farrell, A.P. & Richards, J.G. Defining hypoxia: An integrative synthesis of the response of fish to hypoxia. In *Fish Physiology*, 27 (Richards, J.G., Farrell, A.P., Brauner, C.J. eds), pp 487-503. Amsterdam: Elsevier (2009).
36. Frey, D. G. (1971) Worldwide distribution and ecology of *Eurycercus* and *Saycia* (*Cladocera*). *Limnol. Oceanogr.* **16**, 254.
37. MacKenzie, B. R. & Schiedek, D. Daily ocean monitoring since the 1860s shows record warming of northern European seas. *Glob. Change Biol.* **13**, 1335–1347. (2007).
38. Oeberst, R. *et al.* When is year-class strength determined in western Baltic herring? *ICES J. Mar. Sci.* **66**, 1667-1672 (2009).
39. Polte, P., Kotterba, P., Hammer, C. & Gröhsler, T. Survival bottlenecks in the early ontogenesis of Atlantic herring (*Clupea harengus*, L.) in coastal lagoon spawning areas of the western Baltic Sea. *ICES J. Mar. Sci.* **71**, 982-990 (2014).
40. Simm, M. & Ojaveer, E. Dynamics of Copepods and Fish Larvae in Pärnu Bay (NE Part of the Gulf of Riga) in the Spring-Summer Period. *Proc. Estonian Acad. Sci. Biol. Ecol.* **49**, 317–326 (2000).
41. Ojaveer, E. & Simm, M. Effect of Zooplankton Abundance and Temperature on Time and Place of Reproduction of Baltic Herring Groups. *Merentutkimuslait. Julk./Havsforskningsinst. Skr*. **239**, 139–145 (1975).

42. Kiørboe, Th., Munk, P. & Støttrup, J.G. First feeding by larval herring *Clupea harengus* L. *Dana.* **5**, 95–107 (1985).
43. Brenning, U. Das Zooplankton des Greifswalder Boddens. *Meer und Museum* **5**, 36–43 (1989).
44. Fennel, W. & Neumann, Th. Variability of copepods as seen in a coupled physical-biological model of the Baltic Sea. *ICES Mar. Sci. Symp.* **219**, 208–219 (2003).
45. Moll, A. & Stegert, C. Modelling *Pseudocalanus elongates* stage-structured population dynamics embedded in a water column ecosystem model for the northern North Sea. *J. Mar. Syst.* **64**, 35–46 (2007).
46. Blaxter, J.H.S. & Hempel, G. Biologische Beobachtungen bei der Aufzucht von Heringsbrut. *Helgoländer Wiss. Meeresunters. VII* **5**, 260–283 (1961).
47. Klinkhardt, M. Gedanken zur Abhängigkeit der Laichentwicklung Rügenscher Frühjahrsheringe (*Clupea harengus* L.) von Umweltparametern. *Fisch.-Forsch*. **24**, 22–27 (1986).
48. Cushing, D. H. Plankton production and year-class strength in fish populations: an update of the match/mismatch hypothesis. *Adv. Mar. Biol.* **26**, 249-293 (1990).
49. Sundby, S. Recruitment of Atlantic cod stocks in relation to temperature and advection of copepod populations. *Sarsia* **85**, 277-298 (2000).
50. Righton, D. A *et al.* Thermal niche of Atlantic cod *Gadus morhua*: Limits, tolerance and optima. *Mar. Ecol. Progr. Ser.* **420**, 1-13 (2010).
51. Cheung, W.L., R. Watson, R. & Pauly, D. Signature of ocean warming in global fisheries catch. *Nature* **497**, 365-368 (2013).
52. Sergeant, D. E. Harp seals, man and ice. *Canadian special publication of fisheries and aquatic sciences/Publication speciale Canadienne des sciences halieutiques et aquatiques.* (1991).
53. Nilssen, K. T., Pedersen, O. P., Folkow, L. P. & Haug, T. Food consumption estimates of Barents Sea harp seals. *NAMMCO Sci. Publ.* **2**, 9-27 (2004).
54. Lawson, J. W., Anderson, J. T., Dalley, E. L. & Stenson, G. B. Selective foraging by harp seals *Phoca groenlandica* in nearshore and offshore waters of Newfoundland, 1993 and 1994. *Mar. Ecol. Progr. Ser.* **163**, 1-10 (1998).
55. Enghoff, I. B., MacKenzie, B. R., & Nielsen, E. E. The Danish fish fauna during the warm Atlantic period (ca. 7000–3900 bc): Forerunner of future changes? *Fish. Res.* **87**, 167-180 (2007).
56. Enghoff, I.B. Fishing in Denmark during the Ertebølle period. *Int. J. Osteoarch*. **4**, 65–96 (1994).
57. Schmölcke, U. Holocene environmental changes and the seal (*Phocidae*) fauna of the Baltic Sea: coming, going and staying. *Mammal Rev.* **38**, 231-246 (2008).
58. Storå, J. Sealing and animal husbandry in the Ålandic Middle and Late Neolithic. *Fennosc. Archaeol.* **17**, 57-81 (2000).
59. Storå, J., & Ericson, P. G. A prehistoric breeding population of harp seals (*Phoca groenlandica*) in the Baltic Sea. *Mar. Mammal Sci.* **20**, 115-133 (2004).
60. Ukkonen, P. The early history of seals in the northern Baltic. In *Annales Zoologici Fennici,* pp. 187-207. Finnish Zoological and Botanical Publishing Board (2002).
61. Bennike, O., Rasmussen, P., & Aaris-Sørensen, K. I. M. The harp seal (*Phoca groenlandica* Erxleben) in Denmark, southern Scandinavia, during the Holocene. *Boreas* **37**, 263-272 (2008).
62. Glykou, A. Late Mesolithic-Early Neolithic Sealers: a case study on the exploitation of marine resources during the Mesolithic-Neolithic transition in the south-western Baltic Sea. *Internet Archaeology*, 37 (2014).
63. Fischer, A. Coastal fishing in Stone Age Denmark—evidence from below and above the present sea level and from human bones. *Shell Middens in Atlantic Europe*, 54-69 (2007).
64. Hartz, S., Lübke, H. Terberger, T. From fish and seal to sheep and cattle: new research into the process of neolithisation in northern Germany. *Proc. British Acad.* **144**, 567-594 (2007).
65. Sørensen, L. & Karg, S. The expansion of agrarian societies towards the north–new evidence for agriculture during the Mesolithic/Neolithic transition in Southern Scandinavia. *J. Archaeol. Sci.* **51**, 98-114 (2014).
66. Schmölcke, U. *et al.* Changes of sea level, landscape and culture: A review of the south-western Baltic area between 8800 and 4000BC. *Palaeogeogr. Palaeoclimatol. Palaeoecol.* **240**, 423-438 (2006).
67. Bonsall, C., Cook, G. T., Pickard, C., McSweeney, K., Bartosiewicz, L. Dietary trends at the Mesolithic–Neolithic transition in northwest Europe. In: *Chronology and evolution in the Mesolithic of northwest Europe*, P Crombé, M. van Strydonck, J. Sergant, M Boudin and M Bats (Eds.), pp. 539-562, Cambridge Scholars Publishing, Newcastle upon Tyne (2009).
68. Ritchie, K. C., Gron, K. J., & Price, T. D. Flexibility and diversity in subsistence during the late Mesolithic: Faunal evidence from Asnæs Havnemark. *Danish J. Archaeol.* **2**, 45-64 (2013).
69. Lewis, J.P et al. The shellfish enigma across the Mesolithic-Neolithic transition in southern Scandinavia. *Quatern. Sci. Rev.* **151**, 315-320 (2016).
70. Storå, J. & Lõugas, L. Human exploitation and history of seals in the Baltic during the Late Holocene. *The exploitation and cultural importance of sea mammals*, 95-106 (2005).

71. Spärck, R. Undersøgelser over Østersens (*Ostrea edulis*) Biologi i Limfjorden, særlig med Henblik paa Temperaturens Indflydelse paa Kønsskiftet. Centraltrykkeriet, K_benhavn, 82 pp. (1924).
72. Jensen, A. S. & Spärck, R. Bl_ddyr II. Saltvandsmuslinger. Danmarks Fauna **40**, 1–208 (1934).
73. Enghoff, I. B. Mesolithic eel-fishing at Bjørnsholm, Denmark, spiced with exotic species. *J. Danish Archaeol.* **10**, 105-118 (1991).
74. Nuñez, M. & Storå, J. Shoreline chronology and economy in the Åland Archipelago 6500-4000 bp. In *PACT* (No. 36, pp. 143-161). Conseil de l'Europe (1991).
75. Milner, N. Oysters, cockles and kitchen middens: Changing practices at the Mesolithic/Neolithic transition. *Consuming passions and patterns of consumption*, 89-96 (2002).
76. Bronk Ramsey, C. Bayesian analysis of radiocarbon dates. *Radiocarbon* **51**, 337-360 (2009).
77. Reimer, P. J. *et al.* IntCal13 and marine13 radiocarbon age calibration curves 0-50,000 years cal BP. *Radiocarbon* **55**, 1869-1887 (2013).
78. Rosentau, A., Harff, J., Oja, T. & Meyer, M. Postglacial rebound and relative sea level changes in the Baltic Sea since the Litorina transgression. *Baltica* **25,** 113–120 (2012).
79. Seifert, T., B. Kayser, and F. Tauber. "Bathymetry data of the Baltic Sea." http://www.io-warnemuende.de/topography-of-the-baltic-sea.html.

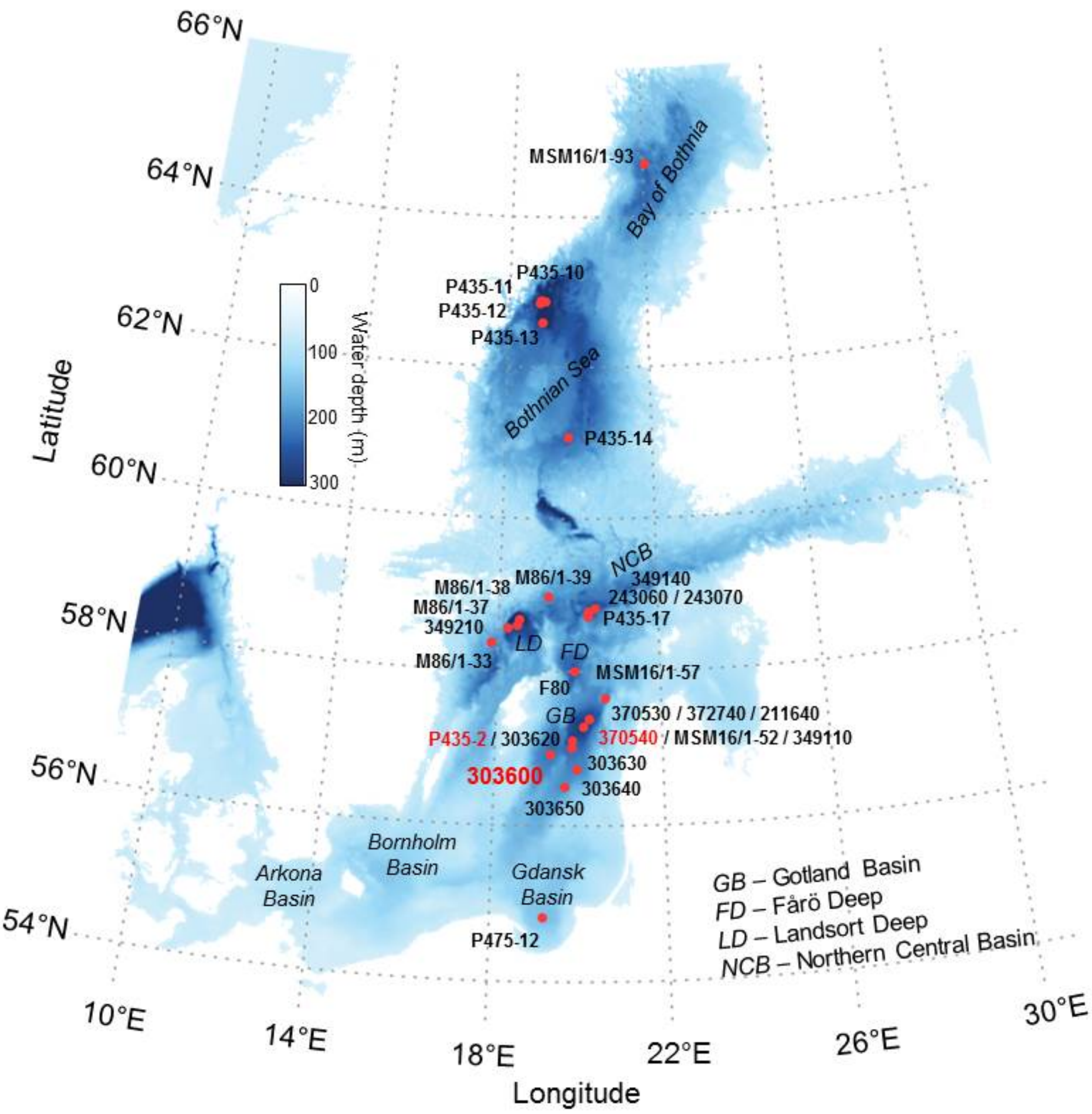

Fig. S1: Sediment coring sites in the Baltic Sea (Paleo-bathymetry at c. 6,500 cal. BP). For detailed site location see Table S1. The map was created using the software package GrADS 2.1.1.b0 (http://cola.gmu.edu/grads/), using published bathymetry data[78,79].

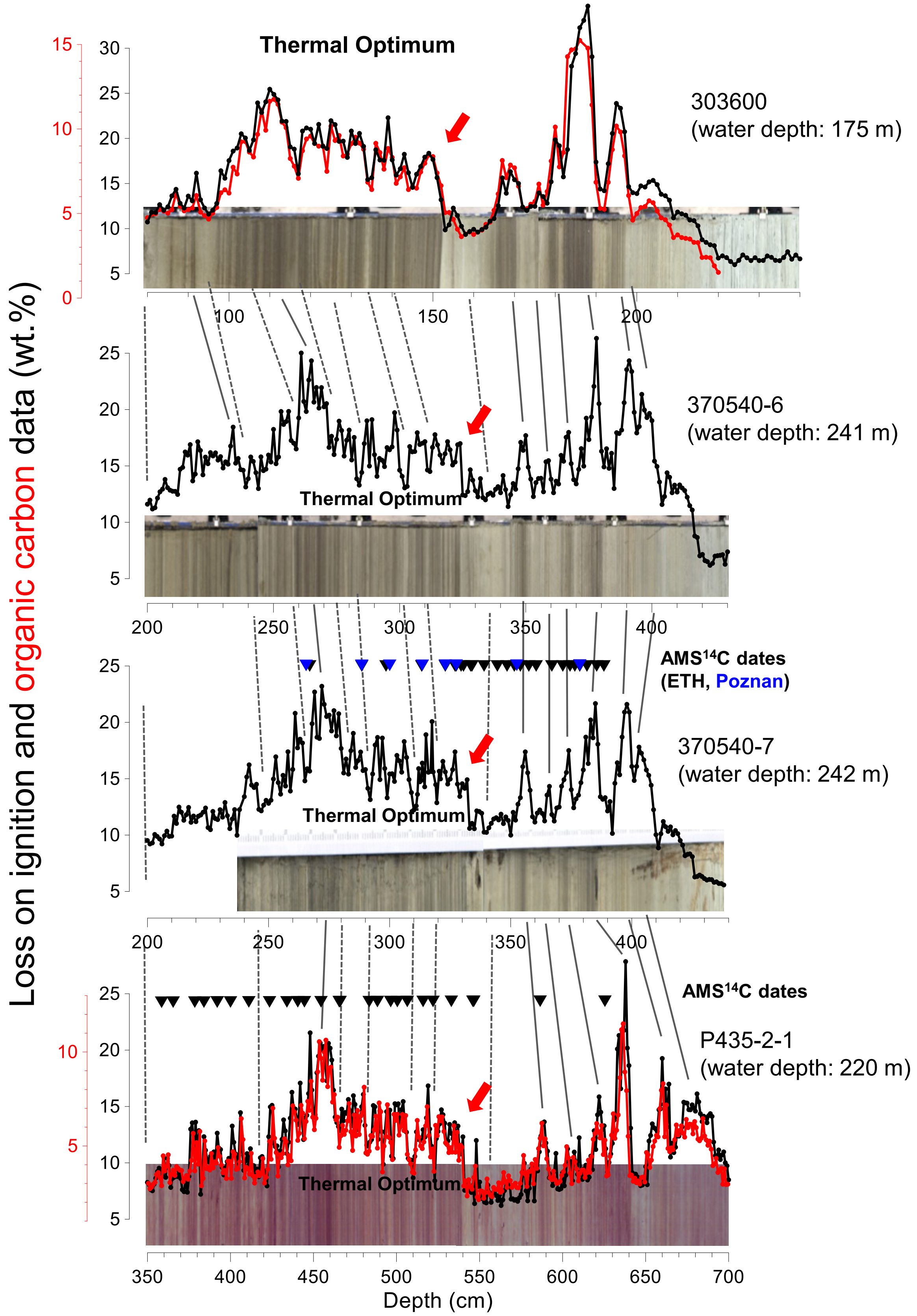

Fig. S2: Correlation of organic carbon (red curves) and LOI (black curves) data from the key-cores (dashed grey lines: characteristic minima; grey lines: characteristic maxima). The chronology was developed based on 69 AMS ^{14}C dates of benthic foraminifera in core P435-2-1 and 370540-7. Depth positions of AMS ^{14}C dates are marked (black triangles: ETH Zürich, blue triangles: Poznan). The age-depth relationships were transferred onto core 303600 correlating the high-resolution LOI and TOC profiles.

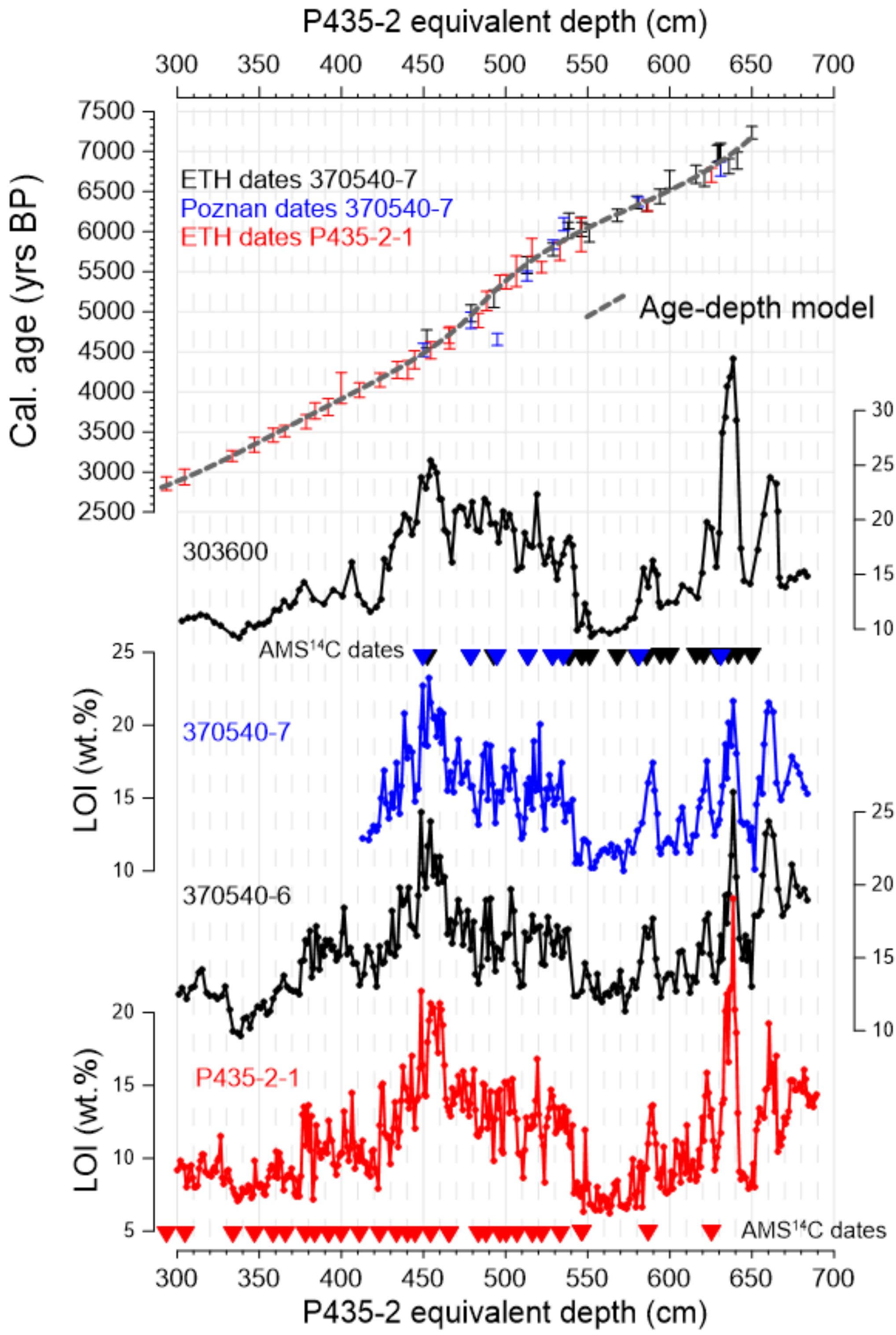

Fig. S3: Loss on ignition (LOI) records of 370540-6(-7) and 303600 were correlated to core P435-2-1 and put on P435-2-1 equivalent depth scale. AMS ^{14}C dates of benthic foraminifera in cores 370540-7 and P435-2-1 (blue triangles: ETH dates, blue: Poznan dates), calibrated ages (upper graph) are shown vs. P435-2-1 equivalent depth scale. The resulting age model is a polynomial fit (upper graph).

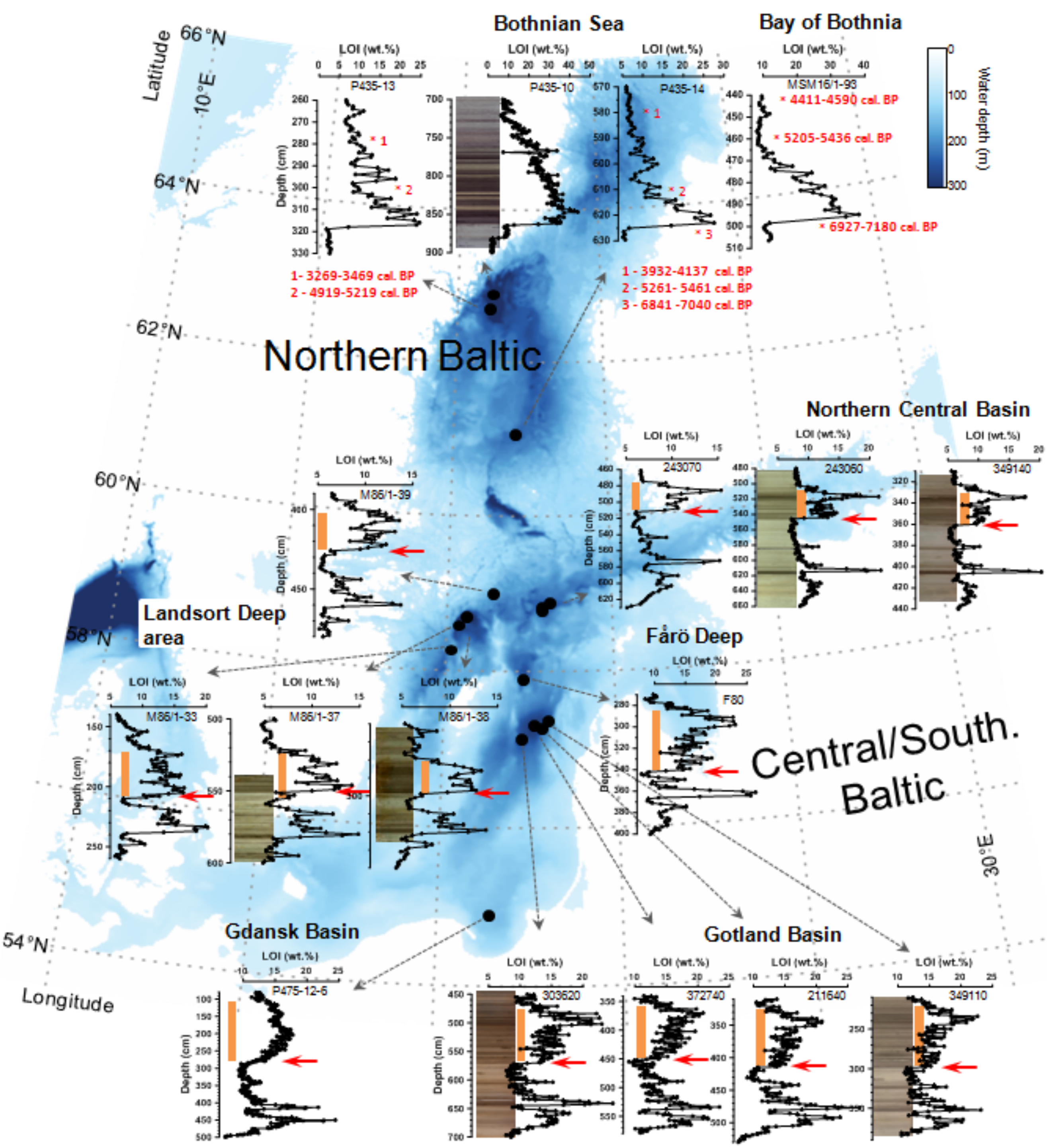

Fig. S4: Loss on ignition (LOI) profiles vs. depth from various sites from the entire deeper Paleo-Baltic. The sharp transition (red arrow) from a marked cold (oxic, low LOI, homogenous sediments) to a warm phase (hypoxic, high LOI, laminated sediments) at c. 6,000 cal. BP is clearly seen in all records from the central and southern Baltic. Only the deeper part of the northern Baltic (upper panel) was hypoxic before 6,000 cal. BP. During the Thermal Optimum (vertical orange bar) the entire Baltic was hypoxic but conditions gradually changed to oxic after the Optimum. Records from the key-sites (Extended data Figure 2) are not included here but fit these LOI profiles very well. AMS^{14}C are marked in profiles from the northern cores. The map was created using the software package GrADS 2.1.1.b0 (http://cola.gmu.edu/grads/), using published bathymetry data[78,79].

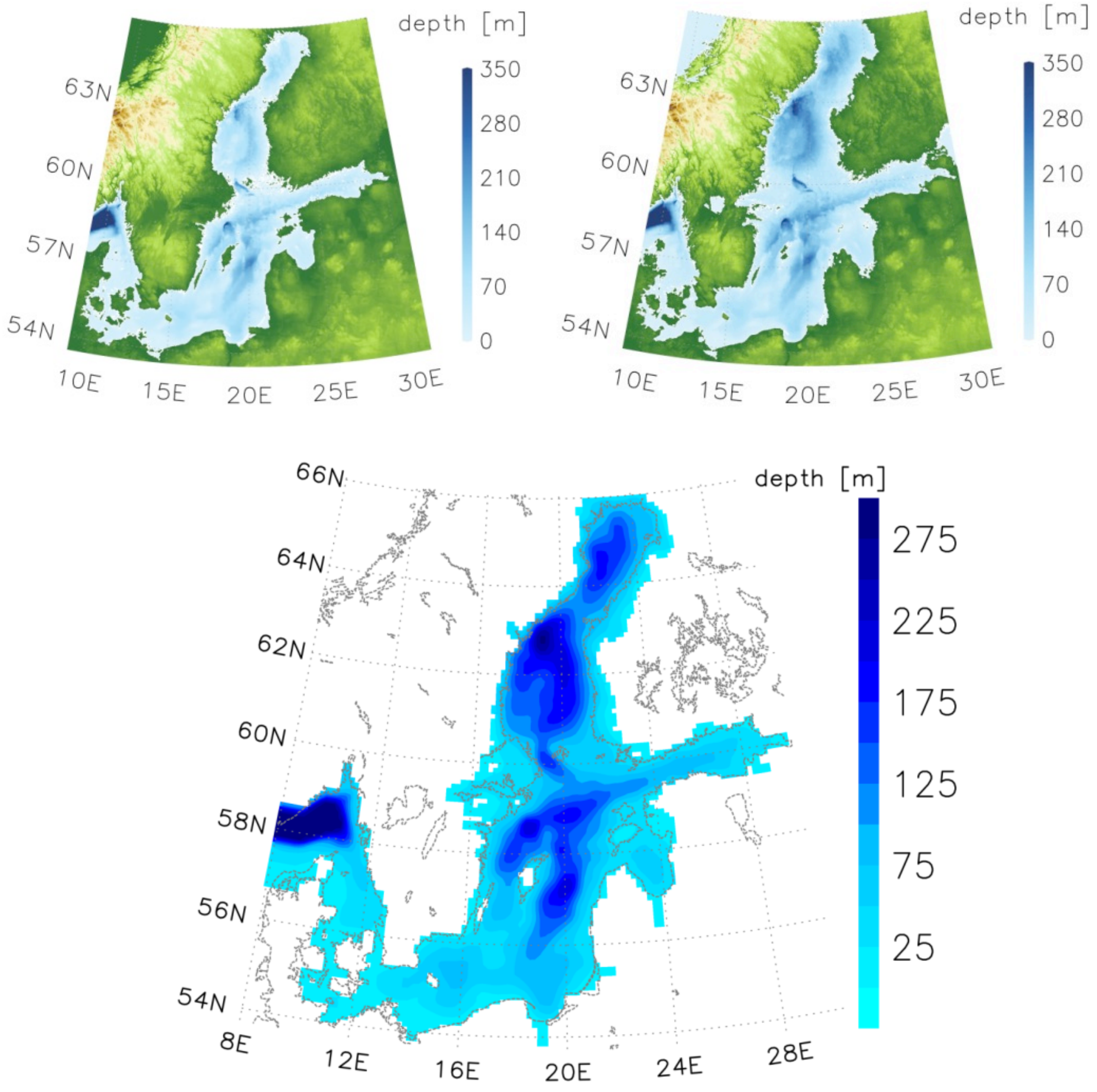

Fig. S5: Reconstructed contemporary (**A**) and paleo- (**B** - 6,500 cal. yr BP) bathymetry for the Baltic Sea. About 6,500 cal. yr BP the northern Baltic Sea basins were more than 100 m deeper than today. The southern Baltic remained nearly unchanged. The derived paleobathymetry was projected onto the 6 nautical miles model grid and is shown in (**C**). Note that the contemporary coastline is marked by a dashed grey line in (**C**). The map was created using the software package GrADS 2.1.1.b0 (http://cola.gmu.edu/grads/), using published bathymetry data[78,79].

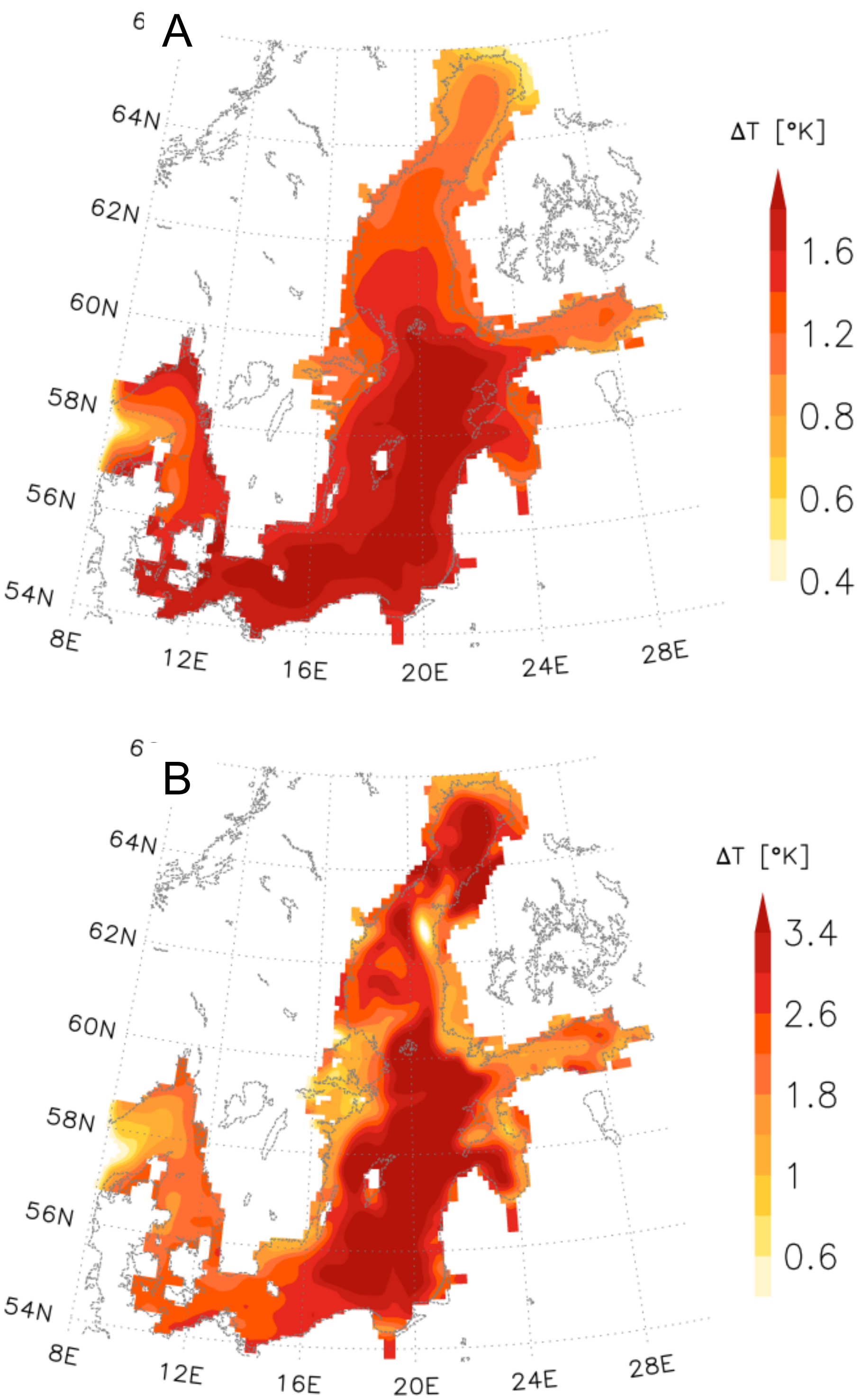

Fig. S6: Modeled mean annual (A) and summer (JJA, B) temperature difference between cold and warm climate in the upper 15 m surface waters using the delta change approach. The map was created using the software package GrADS 2.1.1.b0 (http://cola.gmu.edu/grads/), using published bathymetry data[78,79].

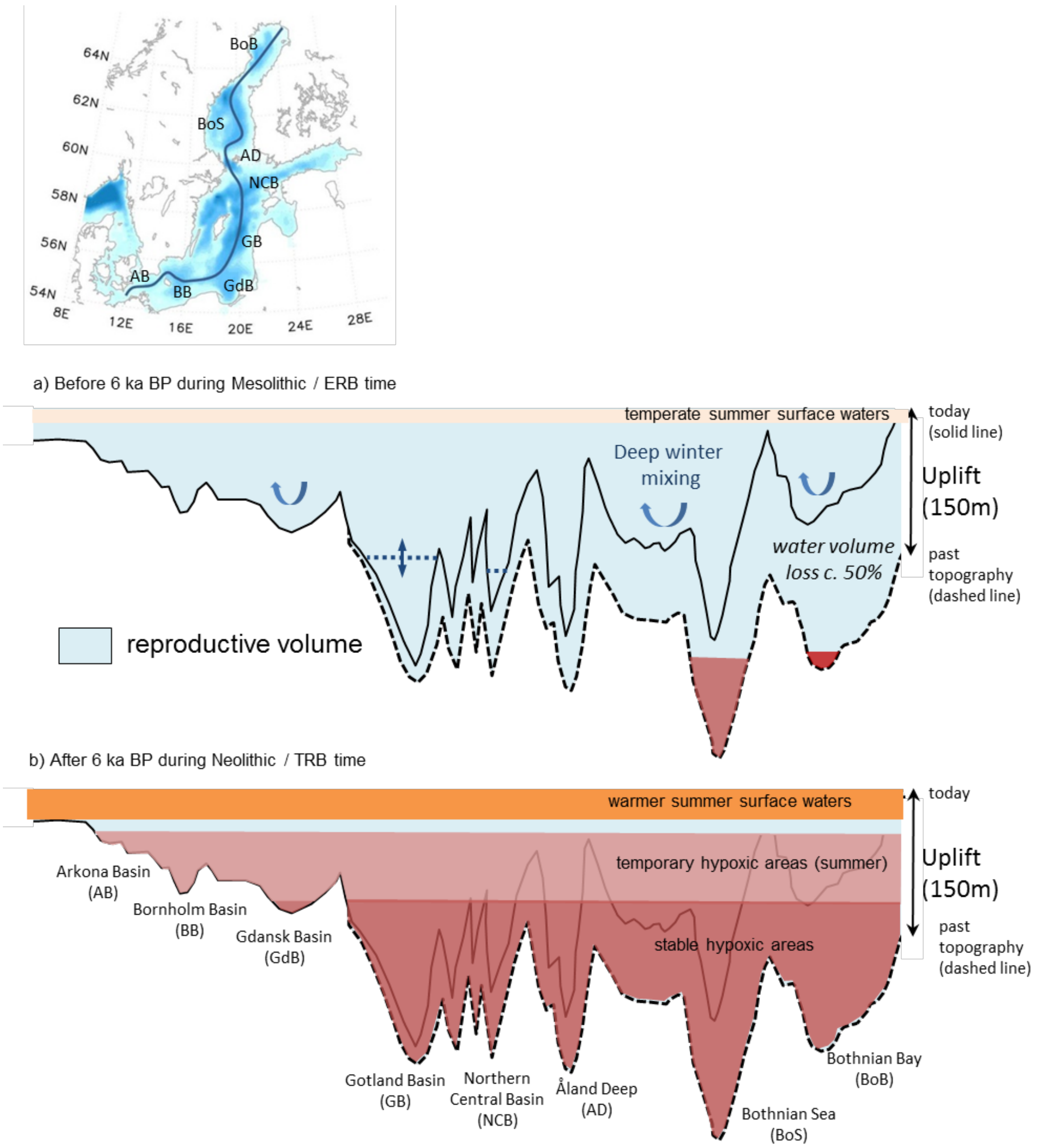

Fig. S7: Schematic changes in the reproductive volume (light blue area) for cod in the Baltic Sea (a) before and (b) after 6,000 cal yr BP. Note the large decrease in reproductive volume which is assumed to have caused a major reduction in maritime resources resulting from the warming and consequent stratification. This may have played a role (in addition to improving conditions for agriculture) in the observed cultural shift. The cross section of the Baltic is along the transect indicated in the map. The map and cross sections were created using the software package GrADS 2.1.1.b0 (http://cola.gmu.edu/grads/), using published bathymetry data[78,79].

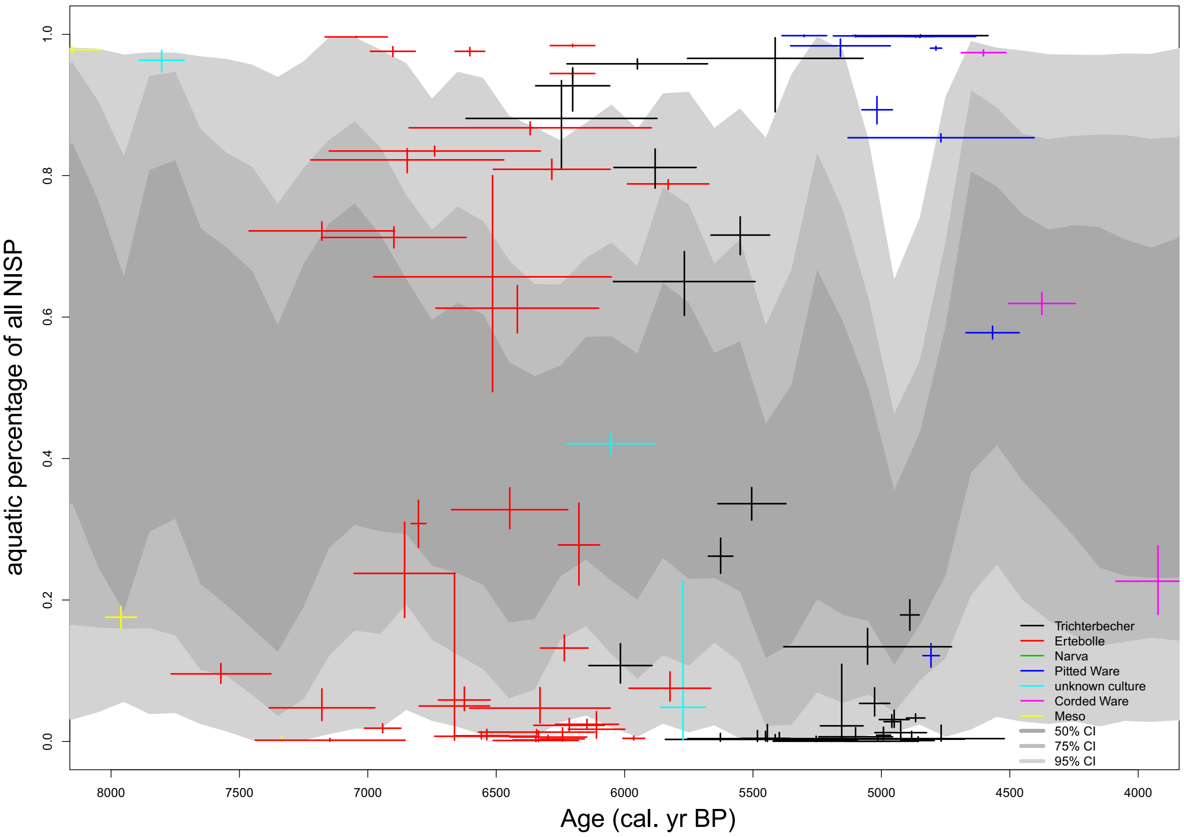

Fig. S8. Number of identified specimens (NISP) of aquatic species as a percentage of the total NISP for archaeological site-phases close to the coast in the polygon area shown in Fig 1. Crosses represent the chronological range (1 sigma CI on x-axis) and aquatic percentage of all NISP (95% CI on y-axis). The grey envelopes represent the 95%, 75% and 50% CI of a rolling mean across all site-phases. These broad CI envelopes incorporate chronological uncertainty, NISP proportion uncertainty, and sampling uncertainty from periods with a small number of site-phases. Calculation methods are detailed in the SI.

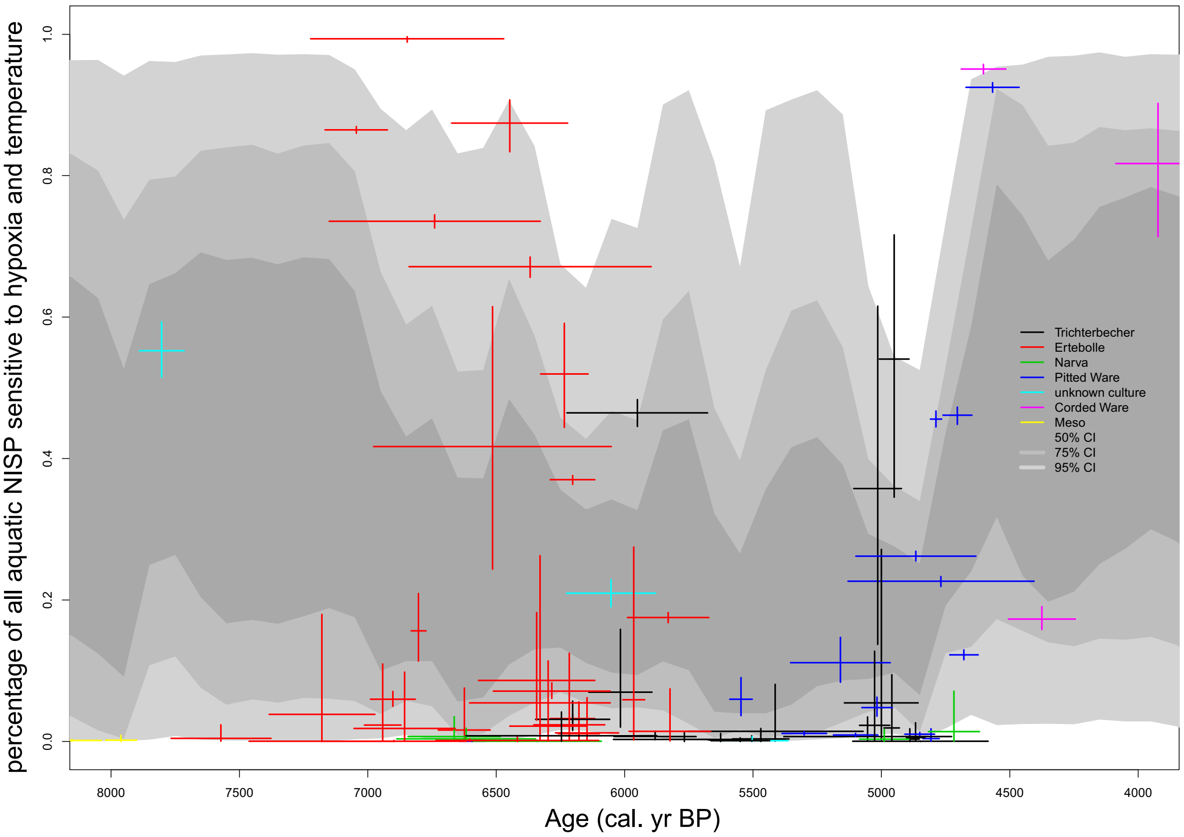

Fig. S9. Number of identified specimens (NISP) of aquatic species sensitive to hypoxia and increased temperature as a percentage of the total aquatic NISP for archaeological site-phases close to the Baltic coast. Crosses represent the chronological range (1 sigma CI on x-axis) and percentage of aquatic species influenced by temperature change and hypoxia (95% CI on y-axis). The grey envelopes represent the 95%, 75% and 50% CI of a rolling mean across all site-phases. These broad CI envelopes incorporate chronological uncertainty, NISP proportion uncertainty, and sampling uncertainty from periods with a small number of site-phases. Calculation methods are detailed in the SI.

Table S1: AMS ^{14}C dating results and calibrated ages (one sigma range, using Marine13 in Calib 7.01 program) from Gotland Basin key-site P435-2-1 and 370540-7, and other sites of the central and northern Baltic Sea. Core 370540-7: (*) denotes equivalent core depth in gravity core P435-2-1. Samples were measured as gas (ETH) and graphite samples (Poz), respectively.

Depth (cm) in core	Lab code	Material dated	^{14}C age	Calibrated age 1σ
P435-2-1, Gotland Basin (57°06.33´N, 19°50.77´E, water depth: 218 m)				
293-294	ETH-55053	*Elphidium* spp.	3090 ± 70	2770 – 2940
304-305	ETH-55054	*Elphidium* spp.	3150 ± 70	2840 – 3040
331-336	ETH-56921	*Elphidium* spp.	3345 ± 50	3130 – 3270
346-348	ETH-56922	*Elphidium* spp.	3465 ± 75	3240 – 3430
358-359	ETH-56231	*Elphidium* spp.	3570 ± 75	3380 – 3550
365-366	ETH-55057	*Elphidium* spp.	3615 ± 55	3440 – 3580
378-379	ETH-56923	*Elphidium* spp.	3710 ± 65	3540 – 3720
384	ETH-59705	*Elphidium* spp.	3815 ± 75	3660 – 3860
392	ETH-59706	*Elphidium* spp.	3860 ± 75	3710 – 3920
399-401	ETH-56233	*Elphidium* spp.	4030 ± 140	3860 – 4240
410-412	ETH-56924	*Elphidium* spp.	4010 ± 65	3930 – 4110
423-424	ETH-55059	*Elphidium* spp.	4095 ± 55	4060 – 4230
434	ETH-59707	*Elphidium* spp.	4185 ± 75	4170 – 4380
440-441	ETH-56925	*Elphidium* spp.	4190 ± 80	4160 – 4390
444-445	ETH-56926	*Elphidium* spp.	4290 ± 80	4290 – 4520
454-455	ETH-56927	*Elphidium* spp.	4375 ± 75	4410 – 4620
465-466	ETH-56928	*Elphidium* spp.	4500 ± 70	4610 – 4800
466	ETH-59708	*Elphidium* spp	4490 ± 110	4540 – 4810
473-474	ETH-55060	*Elphidium* spp.	4445 ± 65	4520 – 4720
482.5-484	ETH-56929	*Elphidium* spp.	46607 ± 75	4800 – 4980
488-489	ETH-56930	*Elphidium* spp.	4826 ± 80	5020 – 5250
496-497	ETH-55061	*Elphidium* spp.	5025 ± 75	5290 – 5460
500-501	ETH-56931	*Elphidium* spp.	5020 ± 80	5280 – 5460
505-508	ETH-56242	*Elphidium* spp.	5170 ± 170	5310 – 5700
516	ETH-59709	*Elphidium* spp.	5440 ± 100	5690 – 5920
521-523	ETH-56932	*Elphidium* spp.	5205 ± 65	5480 – 5630
533	ETH-59710	*Elphidium* spp.	5380 ± 110	5640 – 5870
545-547	ETH-56245	*Elphidium* spp.	5570 ± 190	5750 – 6170
586-587	ETH-55064	*Elphidium* spp.	5895 ± 65	6260 – 6380
625-626	ETH-55066	*Elphidium* spp.	6250 ± 70	6610 – 6790
370540-7, Gotland Basin (57°17.01´N, 20°07.25´E; water depth: 243 m)				
264-267 (450*)	Poz-73943	*Elphidium* spp.	4390 ± 50	4440 – 4610
266.5-267.5 (452*)	ETH-62230	*Elphidium* spp.	4450 ± 80	4550 – 4660
288-289 (479*)	Poz-73946	*Elphidium* spp.	4650 ± 80	4790 – 4990
288-289 (479*)	ETH-62231	*Elphidium* spp.	4755 ± 65	4880 – 5090
298-299 (493*)	ETH-62232	*Elphidium* spp.	4860 ± 75	5050 – 5270
299-301 (495*)	Poz-73944	*Elphidium* spp.	4470 ± 75	4580 – 4730
312.5-314.5 (513*)	Poz-73945	*Elphidium* spp.	5080 ± 75	5380 – 5510
313-313.5 (513*)	ETH-62233	*Elphidium* spp.	5230 ± 95	5480 – 5690
322-324 (529*)	Poz-73947	*Elphidium* spp.	5460 ± 75	5780 – 5890
322-324 (529*)	ETH-62234	*Elphidium* spp.	5395 ± 60	5700 – 5850
327 (538*)	ETH-59688	*Elphidium* spp.	5620 ± 85	5920 – 6120
327-328 (538.5*)	ETH-61284	*Elphidium* spp.	5725 ± 80	6030 – 6230

327-328 (538.5*)	Poz-70380	*Elphidium* spp.	5690 ± 60	6010 – 6170
333-334 (546*)	ETH-61286	*Elphidium* spp.	5690 ± 75	6000 – 6180
334 (546.5*)	ETH-59690	*Elphidium* spp.	5635 ± 70	5940 – 6120
338-340 (551*)	ETH-59691	*Elphidium* spp.	5565 ± 85	5870 – 6080
348-349 (568*)	ETH-59693	*Elphidium* spp.	5785 ± 70	6130 – 6280
351-352 (580*)	ETH-61287	*Elphidium* spp.	5960 ± 75	6290 – 6440
352.5 (581*)	Poz-68833	*Elphidium* spp.	5970 ± 50	6310 – 6430
354 (586*)	ETH-59694	*Elphidium* spp.	5900 ± 70	6260 – 6320
357-358 (594*)	ETH-59695	*Elphidium* spp.	6025 ± 75	6350 – 6530
360-361 (600*)	ETH-59696	*Elphidium* spp.	6210 ± 90	6540 – 6760
366-368 (616*)	ETH-59698	*Elphidium* spp.	6255 ± 90	6600 – 6830
371-372 (621*)	ETH-59699	*Elphidium* spp.	6220 ± 75	6570 – 6760
374-375 (629*)	ETH-61288	*Elphidium* spp.	6470 ± 75	6870 – 7080
376-377 (630*)	ETH-59700	*Elphidium* spp.	6475 ± 75	6880 – 7080
378-379 (631*)	ETH-61289	*Elphidium* spp.	6485 ± 80	6890 – 7100
378.5 (631*)	Poz-70381	*Elphidium* spp.	6320 ± 70	6700 – 6870
381.5 (636*)	ETH-59701	*Elphidium* spp.	6350 ± 75	6720 – 6910
385.5 (641*)	ETH-59702	*Elphidium* spp.	6415 ± 80	6780 – 7000
388.5 (650*)	ETH-59703	*Elphidium* spp.	6710 ± 75	7160 – 7310
P435-13-4, Bothnian Sea (62°35.18´N, 18°58.13´E; water depth: 215 m)				
282-285.5	ETH-56905	*Elphidium* spp.	3495 ± 80	3270 – 3470
303.5-305.5	ETH-56907	*Elphidium* spp.	4780 ± 110	4920 – 5220
P435-14-2, Bothnian Sea (61°03.98´N, 19°43.13´E; water depth: 141 m)				
578-580	ETH-59736	*Elphidium* spp.	4020 ± 70	3930 – 4140
610-611	ETH-59738	*Elphidium* spp.	5000 ± 85	5260 – 5460
624-625	ETH-59739	*Elphidium* spp.	6450 ± 70	6840 – 7040
MSM16/1-93, Bay of Bothnia (64°42.00´N 22°03.72´E; water depth: 130 m)				
441.5-442.5	ETH-56908	*Elphidium* spp.	4365 ± 65	4410 – 4590
464.5	ETH-59704	*Elphidium* spp.	4955 ± 80	5210 – 5440
495.5-497.5	ETH-56909	*Elphidium* spp.	6545 ± 110	6930 – 7180
